# The ACCE method: an approach for obtaining quantitative or qualitative estimates of residual confounding that includes unmeasured confounding

**DOI:** 10.12688/f1000research.4801.2

**Published:** 2015-04-29

**Authors:** Eric G. Smith

**Affiliations:** 1 Psychiatrist, The Center for Organizational and Implementation Research (CHOIR) and the Mental Health Service Line of the Department of Veterans Affairs, Edith Nourse Rogers Memorial Medical Center, Bedford, MA 01730, USA; 2 Departments of Psychiatry and Quantitative Health Sciences, University of Massachusetts Medical School, Worcester, MA 01655, USA

**Keywords:** observational study, nonrandomized study, intervention research, residual confounding, unmeasured confounding, bias amplification, confounding amplification, propensity scores

## Abstract

**Background:**  Nonrandomized studies typically cannot account for confounding from unmeasured factors.

**Method:**  A method is presented that exploits the recently-identified phenomenon of  “confounding amplification” to produce, in principle, a quantitative estimate of total residual confounding resulting from both measured and unmeasured factors.  Two nested propensity score models are constructed that differ only in the deliberate introduction of an additional variable(s) that substantially predicts treatment exposure.  Residual confounding is then estimated by dividing the change in treatment effect estimate between models by the degree of confounding amplification estimated to occur, adjusting for any association between the additional variable(s) and outcome.

**Results:**  Several hypothetical examples are provided to illustrate how the method produces a quantitative estimate of residual confounding if the method’s requirements and assumptions are met.  Previously published data is used to illustrate that, whether or not the method routinely provides precise quantitative estimates of residual confounding, the method appears to produce a valuable qualitative estimate of the likely direction and general size of residual confounding.

**Limitations:**  Uncertainties exist, including identifying the best approaches for: 1) predicting the amount of confounding amplification, 2) minimizing changes between the nested models unrelated to confounding amplification, 3) adjusting for the association of the introduced variable(s) with outcome, and 4) deriving confidence intervals for the method’s estimates (although bootstrapping is one plausible approach).

**Conclusions:**  To this author’s knowledge, it has not been previously suggested that the phenomenon of confounding amplification, if such amplification is as predictable as suggested by a recent simulation, provides a logical basis for estimating total residual confounding. The method's basic approach is straightforward.  The method's routine usefulness, however, has not yet been established, nor has the method been fully validated. Rapid further investigation of this novel method is clearly indicated, given the potential value of its quantitative or qualitative output.

## Introduction

Confounding is a central challenge for virtually all nonrandomized studies. Recent research ^1– 4^ has revealed that propensity score methods or other highly-multivariate methods may actually significantly increase, or “amplify,” the residual confounding remaining after their application. Understandably, this recently-recognized property of propensity score methods and other highly-multivariate methods have been generally viewed as a limitation or complication to their use. More recently, however, a study has indicated that the degree of confounding amplification (also termed “bias amplification” ^4^) occurring between propensity score models appears to be *quantitatively predictable* (at least in simulation) ^5^. This quantitative predictability of confounding amplification may also be suggested by more theoretical presentations of confounding amplification ^4^.

Not yet recognized, to my knowledge, is the *extremely* valuable corollary that results: the predictability of confounding amplification should, in principle, permit extrapolation back to an unamplified value of the total residual confounding originally present. (Throughout this manuscript “confounding” refers to baseline confounding. Confounding occurring after treatment initiation from differential discontinuation of the intervention in the treatment group of interest versus the comparison group is not addressed in this manuscript. A possible approach to estimating confounding occurring after treatment initiation, based on the same principles described here, is briefly discussed in Appendix 2.3b). In this manuscript and the associated appendices, I describe the general framework and detailed specifics of a new method designed to use amplified confounding to estimate total residual confounding (including from unmeasured factors), and thus provide an unconfounded treatment effect estimate.

The basic logic of this method is straightforward, but its performance in practice has yet to be confirmed. Testing of this method on both simulated and real-world data is clearly needed. This manuscript does illustrate, however, that even when this method is not able to provide a precise quantitative estimate of residual confounding, it may provide a very useful qualitative estimate of the likely direction and general size of residual confounding. This manuscript provides detailed information to the research community intended to facilitate the rapid evaluation of the performance of this method when applied to simulated and real-world data.

## Method

This four-step method deliberately amplifies confounding to permit estimation of unmeasured confounding. This estimate is then subtracted, along with the measured confounding of the variable or variables producing the amplification, from the original treatment effect estimate. This approach of deliberately amplifying confounding initially may seem counterintuitive. The text below seeks to explain the steps of the method (and their rationale) and follows in parallel to their mathematical description in the Appendix Table. Ultimately, a single equation incorporating all components of the method is derived. Some readers may also find the largely nonmathematical metaphors provided in Appendix 1 helpful.

### Step 1 – Create nested propensity score models and generate treatment effect estimates

The “Amplified Confounding-based Confounding Estimation (ACCE) Method” depends on the use of two propensity score models, one (“Model 1”) nested in the other (“Model 2”) so that Model 2 contains all the Model 1 covariates plus an additional variable or variables. The variable(s) introduced to produce Model 2 from Model 1 is termed the “Introduced Variable(s).” Importantly, the Introduced Variable(s) should be sufficiently associated with treatment exposure to produce discernible confounding amplification. That is, the Introduced Variable(s) should further predict treatment exposure sufficiently to substantively *increase* differences between the treatment groups in the prevalence of those confounding factors that are not present in either model.

### Step 2 – Estimate both the proportional amplification of confounding and the quantitative change in the treatment effect estimate between Model 1 and Model 2

In principle, the original confounding existing prior to amplification can be estimated by extrapolation backwards if both the proportional amount of confounding amplification and the quantitative change in the treatment effect estimate occurring between two propensity score models can be estimated accurately. To give a very simple example, consider a model (Model 2), which adds a single Introduced Variable compared to the original model (Model 1). This Introduced Variable sufficiently explains treatment exposure that its inclusion is expected to exhibit 50% confounding amplification compared to Model 1. Assume the observed treatment effect risk ratio (RR) changed in this circumstance from 1.10 in Model 1 (a beta coefficient of 0.09531) to approximately 1.15 (a beta coefficient of 0.142965, which is strictly equivalent to an RR of 1.15369). If the Introduced Variable(s) had no association itself with exposure, then the increase in beta coefficient would result entirely from the 50% amplification of confounding (ignoring random variation). Since a 50% increase in the confounding increased the beta coefficient 0.047655, this would imply that the original confounding was 0.047655 / 0.5 = 0.09531. This is enough to account for the entire association with outcome originally attributed to treatment. That is, the observed increase of the effect estimate upon confounding amplification is sufficient to suggest that the entire originally-observed “treatment effect” estimate was in fact due to confounding ( Endnote A).

Attention is needed during the method’s implementation, however, to ensure: 1) that either the Introduced Variable(s) truly does have no association with outcome, or to correct for this Introduced Variable-outcome association if it is present (Steps 3 and 4 of the method); and 2) that changes between the two models distinct from confounding amplification are minimized to the extent feasible ( Appendix 2). In addition, the method requires an ability to estimate the proportional amount of confounding amplification occurring between two propensity score models.

Two very different approaches to estimating proportional confounding amplification suggest themselves. One approach would be to estimate amplification from existing or future simulation research based on particular metrics of exposure prediction. An example of this approach is research published using the linear measure of exposure prediction, R ^2^ ^5^. This work demonstrated that, for propensity score stratification or matching approaches, a linear relationship exists between *unexplained* variance in exposure (i.e., 1 - R ^2^) and the proportional amount of confounding amplification occurring across the range of R ^2^ = 0.04 to 0.56. This simulation study ^5^, using a propensity score based on a linear probability model, also made the important demonstration that different unmeasured confounders appear to be amplified to a highly similar degree. A key assumption of the ACCE Method is that residual confounding attributable to different confounders is uniformly or relatively uniformly amplified in Model 2 compared to Model 1.

There is still a possibility, however, that the mathematical predictability of the proportional amplification based on 1 - R ^2^ that was observed in simulation may be merely a consequence of the particular conditions of that simulation. Other work, however, also suggests the possibility of estimating the proportional amount of confounding amplification through the 1 - R ^2^ relationship ^4^.

Further research is needed to thoroughly confirm that a predictable relationship does indeed exist between predication of exposure as measured by R ^2^ and resulting confounding amplification. Research is also needed to determine if a similarly predictable relationship exists for other metrics of exposure prediction (such as those proposed for logistic regression ^6, 7^). Finally, research is needed to establish whether the apparent nonlinearities between the prediction of exposure and confounding amplification at more extreme ranges of prediction, suggested by some manuscripts ^5^ but not others ^4^, actually do exist.

A second approach to estimating the proportional amplification of confounding between two models would be to adopt an “internal marker” strategy. This strategy consists of deliberately withholding a measured covariate from both models to allow the increase in its imbalance between treatment groups in Model 2 to serve as an approximate indicator of the proportional confounding amplification that has occurred. It is possible, however, that the “internal marker” strategy might consistently yield at least a slight degree of underestimate of the amount of confounding amplification ( Appendix 3.1).

If the “Introduced Variable” is known to be a true instrumental variable, then Steps 1 and 2 are the only steps required. Whether this approach would be any advantage over a conventional instrumental variable regression, however, is uncertain. The next two steps describe the additional calculations necessary to adjust for an association between the Introduced Variable and outcome if the Introduced Variable is not known to be an instrumental variable. These steps add a minor amount of computational complexity to the method, as well as increase the uncertainty concerning the strict quantitative accuracy of the method’s estimates (as discussed below). Importantly, however, these steps also may greatly broaden the method’s applicability, since many more variables with substantial association with exposure (i.e., candidate Introduced Variables) are likely to exist that have some association with outcome than do not.

### Step 3 – Adjust for the association between the Introduced Variable and outcome

In most cases, the addition of a variable(s) to Model 2 will alter the amount of residual confounding present in Model 2 compared to Model 1, *independent* of its effect producing confounding amplification (i.e., it is rare for a variable to have absolutely no association with outcome). The consequence of this is that what is being amplified in Model 2 is not the actual quantity being sought (the total residual confounding in Model 1) but only a fraction of this quantity. Specifically, the quantity being amplified is the fraction of the Model 1 residual confounding separate from that attributable to the Introduced Variable.

Because the Introduced Variable(s) is included in the Model 2 propensity score, the Introduced Variable does not amplify. Not only does the confounding from this variable not amplify, but any contribution to confounding attributable to the Introduced Variable would be generally expected to *decrease* in Model 2 compared to Model 1. This decrease results from the fact that the Introduced Variable will almost certainly become more balanced now that it is included in the propensity score. As a result, when we want to estimate the quantitative change in the treatment effect estimates attributable to amplified confounding, we must first subtract the contribution of the decreased confounding attributable to the change in the balance of the Introduced Variable(s). Arriving at an estimate of the change in treatment effect estimates between Model 2 and Model 1 that is solely attributable to the amplification of confounding between Model 1 and Model 2 is crucial, because this quantity will allow us to extrapolate backwards to an estimate of the residual confounding attributable to all unmeasured confounders except the Introduced Variable(s). Because the Introduced Variable does not amplify, its contribution to residual confounding cannot be estimated through this extrapolation. Instead, its effect must be removed separately. Second, we must also remove the contribution of the Introduced Variable(s) from the original, Model 1 treatment effect estimate to obtain the desired unconfounded treatment effect estimate.

To illustrate the need for the first adjustment (adjusting the change in treatment effect estimate to account for the change attributable to improved balance in the Introduced Variable), consider the following case: the amplification of residual confounders that are unmeasured or nonincluded in *both* Models (which we will term the “amplifiable fraction” of total residual confounding) increases the treatment effect estimate by beta = 0.09531, and insertion of the Introduced Variable into Model 2 changes its confounding by beta = -0.09531. In this case, the observed change in the treatment effect estimate between Model 2 and Model 1 would be zero. However, it would not be correct to conclude that no quantitative change in the Model 2 treatment effect estimate attributable to confounding amplification had occurred. Instead, a sizeable quantitative change due to confounding amplification occurred, but it had simply been concealed by an equal change in the other direction due to reduced confounding from the Introduced Variable. Only by subtracting the change in confounding expected to result from increased balance in the Introduced Variable does the quantitative impact of the confounding amplification become apparent.

The need for the second adjustment exists because the Introduced Variable did not amplify, and thus its contribution to Model 1 confounding will not be included in the back-extrapolation that is performed to estimate the original amount of confounding due to the “amplifiable fraction” (the fraction of confounding that *can* be amplified). The Introduced Variable(s)’s contribution to Model 1 confounding must be directly estimated and removed separately. These two adjustments involving the Introduced Variable(s) usually will be similar in magnitude, but not identical, as explained later.

To make both of these adjustments I propose obtaining a coefficient(s) for the Introduced Variable(s) from regression models of the outcome that include all other propensity score covariates ( Endnote B and Appendix Table Step 3a). This Introduced Variable-outcome regression coefficient can then be inserted into the Bross equation ^8^ is used to estimate the confounding attributable to the Introduced Variable(s) in both Model 1 and Model 2. (The Bross equation ^8^, which recently has been used by Schneeweiss and colleagues in their high-dimensional propensity score algorithm ^9^, quantifies the amount of confounding attributable to a confounder. The Bross equation provides this estimate by combining the strength of the association between the covariate and outcome with the imbalance in the covariate between the treatment groups. Its use is demonstrated in the Appendix Table Step 3b1 and Appendix Table Step 3b2).

This regression-based correction appears, in theory, to be an imperfect solution, but how much these imperfections routinely interfere with the method’s performance is uncertain. The potential imperfections arise from two sources. First, it is plausible that the regression-based coefficient may not fully reflect the sum effect upon confounding that results when the Introduced Variable is inserted into Model 2. If the Introduced Variable is correlated with any unmeasured confounders, then inserting of the Introduced Variable(s) in Model 2 would also be expected to also reduce the imbalance in these other unmeasured confounders (at least relative to their Model 2 imbalance if no correlation existed). Of note, this correlation would also be expected to affect the Introduced Variable(s) regression coefficient: it is well appreciated that in regression models two correlated variables can influence the regression coefficient obtained for each of the variables. Unfortunately, it is not well understood, to my knowledge, whether the effect of correlation in regression alters the regression coefficient in a manner that, when this coefficient is used in the Bross equation, the estimated change in confounding approximates the actual change in confounding resulting from the change in the Introduced Variable(s) and its correlates.

Second, the regression equations used to derive the Introduced Variable(s)-outcome regression coefficient optimally would have the same number of variables within them as the propensity score. Thus, some degree of confounding amplification may also exist in the Introduced Variable(s)-outcome coefficients, although this amplification is likely less than observed for the treatment effect estimate, and possibly less problematic ( Appendix 4).

Using the Bross equation, the estimate of the confounding attributable to the Introduced Variable in Model 1 is then subtracted from estimate of such confounding in Model 2. This produces an estimate of the *change* in the treatment effect estimate between Model 1 and Model 2 that is attributable to increased balance in the Introduced Variable(s) (and potentially, to some degree, its correlates) ( Appendix Table Step 3b3). This estimate then is subtracted from the *overall* change in the treatment effect estimate observed between Model 2 and Model 1 ( Appendix Table Step 3c). The result is an important quantity: the quantitative change in the treatment effect estimate attributable to the proportional confounding amplification that occurred between Model 1 and Model 2 ( Appendix Table Step 3d).

### Step 4 – Calculate the unconfounded treatment effect estimate

The final step involves two substeps. First, divide the final result from Step 3d (the change in the treatment effect estimate from Model 1 to Model 2, adjusted to remove the change produced by increased balance in the Introduced Variable(s) and potentially its correlates) by the proportional amount of confounding amplification occurring between Model 1 and Model 2 ( Appendix Table Step 4a). This proportional confounding amplification can be calculated by the ratio of the proportional confounding amplification of both models relative to a state with no confounding amplification. For example if Model’s R ^2^ = was 0.50, then, based on the 1 - R ^2^ relationship, this model it would be expected to contain 2-fold confounding amplification (1 / (1 - 0.5) = 1 / 0.5 = 2). But since we are comparing Model 2’s treatment effect estimate to Model 1’s treatment effect estimate, rather than to a hypothetical, unobserved model with absolutely no confounding amplification, we instead need to take into account the difference in confounding amplification that occurs *between the models*. In a sense, the comparison of amplification between Model 2 and Model 1 respecifies the Model 2 confounding amplification by quantifying it relative to the “starting point” of the confounding amplification observed in Model 1.

For example, if Model 1 had an R ^2^ of 0.25 (leading to a confounding amplification of 1 / (1 - 0.25) = 1 / 0.75 = 1.33, then the proportional amplification of confounding occurring between the 2-fold confounding amplification in Model 2 and the 1.33-fold confounding amplification in Model 1 would be 2 / 1.33 = 1.5. If the difference between treatment effect estimates reflected confounding amplification of 1.5, this means that the difference in the treatment effect estimate represents 1.5 × the original confounding (or a 50% increase in confounding). Therefore the adjusted treatment effect estimate difference observed between the two models would then need to be multiplied by a factor of 2 (i.e., 1 / 0.5) to extrapolate back to an estimate of the original confounding in Model 1. This factor of 2 can be obtained mathematically by subtracting 1 from the proportional confounding amplification predicted between the models (1 / (1.5 - 1) = 1 / 0.5 = 2). (Determining the ratio of the confounding amplification occurring in each of the two models provides the proportional change in confounding amplification between Model 1 and Model 2; subtracting “1” from this ratio accounts for the fact that if no amplification between the models occurs, the ratio will equal “1”, but the confounding amplification will be “0”).

This calculation derives by extrapolation an estimate of the total residual confounding originally in Model 1, *except* for the confounding attributable to the yet-to-be-inserted Introduced Variable(s), and can be represented by the following mathematical term:

$$\left(\frac{{(\text{TEE}}_{\text{M2}}-{\text{TEE}}_{\text{M1}})-{\text{Conf}}_{{\text{IntV}}_{\Delta \text{(M2}-\text{M1)}}}}{\left(\left(\frac{\frac{1}{\left(1-R_{\text{M2}}^{\text{2}}\right)}}{\frac{1}{\left(1-R_{\text{M1}}^{\text{2}}\right)}}\right)-1\right)}\right)$$

In this overall term, “TEE” refers to the treatment effect estimate of the particular model (subscript “M2” denoting Model 2 and “M1” denoting Model 1), thus the term (TEE _M2_ - TEE _M1_) represents the observed difference in treatment effects between the two models. The term “Conf _IntV _Δ(M2-M1)__” represents the change in confounding attributable to the change in balance in the Introduced Variable in Model 2 compared to Model 1. This term is calculated by use of the multivariate Introduced Variable-outcome regression coefficient and the Bross equation. The terms (1 / (1 - R ^2^_M2_)) and (1 / (1 - R ^2^_M1_)) represent the proportional confounding amplification expected in Model 2 and Model 1. The ratio of this proportional confounding amplification provides the proportional amplification occurring between the two models. Subtracting 1 from this ratio permits the extrapolation, from the adjusted change in treatment effect estimates, of the total residual confounding from the amplifiable fraction of Model 1. An algebraic derivation of this term is provided in Endnote F.

One further term is needed to estimate the total residual confounding in Model 1. To reiterate, the confounding from the Introduced Variable is not subject to amplification in Model 2, since it is now included in the propensity score, unlike the rest of the residual confounding in Model 1. Thus, the Introduced Variable(s)’s contribution to Model 1 residual confounding must be accounted for separately, through use of the Introduced Variable-outcome regression coefficient and the Bross equation ( Appendix Table Step 3b1). Entering the imbalances in the Introduced Variable between the treatment groups that are present in Model 1 (that is, relative to a perfect 50%/50% balance) into the Bross equation provides an estimate of the contribution of the Introduced Variable to the Model 1 residual confounding (Endnote G).

Adding the two components of Model 1 residual confounding (i.e., the estimate of residual confounding from the amplifiable fraction plus the confounding attributable to the original, Model 1 imbalance in the yet-to-be-inserted Introduced Variable(s)) produces the method’s estimate of total residual confounding present in Model 1 ( Appendix Table Step 4b1). This total is then subtracted from the Model 1 treatment effect estimate to produce an estimate of the unconfounded treatment effect ( Appendix Table Step 4b2).

The entire approach to estimating residual confounding using the ACCE Method can be summarized by the following equation:

$$TEE_{M\text{1}}-\left(\frac{{(\text{TEE}}_{\text{M2}}-{\text{TEE}}_{\text{M1}})-{\text{Conf}}_{{\text{IntV}}_{\Delta \text{(M2}-\text{M1)}}}}{\left(\left(\frac{\frac{1}{\left(1-R_{\text{M2}}^{\text{2}}\right)}}{\frac{1}{\left(1-R_{\text{M1}}^{\text{2}}\right)}}\right)-1\right)}\right)-{\text{Conf}}_{{\text{IntV}}_{\text{M1}}}=\begin{array}{c} Unconfounded\\ TEE \end{array}$$

This equation subtracts from the original, Model 1 treatment effect estimate (TEE _M1_) the back-extrapolation term estimating the Model 1 residual confounding from the amplifiable confounding fraction (discussed above), as well as the term Conf _IntV _M1__, which represents the separate contribution of the Introduced Variable(s) to Model 1 residual confounding. Subtracting these terms from the Model 1 treatment effect estimate produces, in general principle, an estimate of the unconfounded treatment effect ( Appendix Table Step 4 and Endnote F).

The accuracy of this unconfounded treatment effect estimate, however, is not yet established. The largest uncertainties in this estimate likely come from several factors, including the basic uncertainty whether the proportional confounding amplification occurring between two models is consistently predictable. Two manuscripts suggest such prediction may be possible ^4, 5^, but certainly a more extensive confirmation of this relationship, for R ^2^ and possibly for other metrics of exposure prediction, would be beneficial. In addition, neither of these manuscripts examined real-world data. Thus, questions remain, such as whether real-world data might contain “constraints” to confounding amplification ( Appendix 3.3). Also pertinent are the two uncertainties discussed in Step 3 concerning the adequacy of the Introduced Variable(s) regression coefficient(s) for performing the necessary adjustments to the Model 2 - Model 1 treatment effect estimate term and to the Model 1 total residual confounding. These uncertainties relate to whether the Introduced Variable-outcome regression coefficient adequately reflect changes that would occur in Model 2 in unmeasured confounders correlated with the Introduced Variable(s), as well as whether this Introduced Variable(s)-outcome regression coefficient(s) would also suffer some confounding amplification. Investigation is also needed into the practical question of whether other differences between the models can be sufficiently minimized to prevent them from producing changes in the Model 2 treatment effect estimate separate from confounding amplification ( Appendix 2). These uncertainties, plus others, are highlighted in Appendix Figure 1b in Appendix 5 and listed as research needs in the Discussion.

Nevertheless, the method’s potential to perform an adjustment for the association between the Introduced Variable(s) and outcome suggests that this method might provide quantitatively or qualitatively useful unconfounded treatment effect estimates when instrumental variable analysis is not possible. Associations between the Introduced Variable and outcome may merely complicate, but not preclude, use of the method. In other words, Introduced Variables may not have to meet the “exclusion restriction” traditionally applied to instrumental variables (i.e., having no correlation with outcome other than exclusively through an association with treatment). However, for optimal performance it may still prove advantageous for the Introduced Variable to meet, or nearly meet, the condition of having no correlation with other confounders, at least with respect to unmeasured confounders.

### Conceptualizing the ACCE Method as consisting of two basic components

Since these steps and substeps may seem somewhat complex initially, it may help to conceptualize the ACCE Method as simply involving two overarching components: 1) attempting to quantify the two contributions to residual confounding in Model 1; and 2) subtracting these estimates of unmeasured confounding from the Model 1 treatment effect estimate.

Component 1 involves several operations: creating models to deliberately amplify confounding, measuring their treatment effect estimates, and dividing the change in the treatment effect estimate (adjusted to remove the effect of the change in confounding attributable to the Introduced Variable(s)) by the predicted change in confounding amplification. This entire process estimates one contribution to Model 1 residual confounding: the Model 1 confounding that was amplified through insertion of the Introduced Variable(s) in the Model 2 propensity score. The separate contribution of the Introduced Variable(s) to Model 1 confounding needs to be estimated. This estimate is achieved by entering the imbalance of the Introduced Variable, and its Introduced Variable-outcome regression coefficient, into the Bross equation. (Using slightly different values, the Bross equation also generates the adjustment mentioned above to the change in the treatment effect estimate).

The second component is much simpler, involving only the summing the two parts of original residual confounding estimate and subtracting this sum from the Model 1 treatment effect estimate.

## Illustrative examples

### Four hypothetical examples

Four hypothetical examples are presented to help illustrate the ACCE Method. (As mentioned previously, largely nonmathematical metaphors to help illustrate the method are provided in Appendix 1).

The first hypothetical example simply fleshes out in more detail the particularly simple case already discussed. A propensity score model with an R ^2^ of 0.25 for the prediction of treatment exposure yields a treatment effect estimate of approximately RR = 1.10 when it is used to compare the treated group to a comparison group by matching or stratification. A second propensity score model is generated by adding a single additional covariate that boosts the overall R ^2^ of the expanded propensity score model to 0.5. This change in R ^2^ leads to a decrease in the unexplained variance of exposure (1 - R ^2^), and, as discussed in Step 4 on the preceding page, a predicted 50% amplification of confounding between the models. This second propensity score model yields a treatment effect estimate of approximately RR = 1.15 ( Endnote C). If the Introduced Variable added to the set of Model 1 covariates to produce Model 2 has no genuine association with outcome (and no association with unmeasured covariates that have an association with outcome), then there is no need to adjust for this association in Steps 3 and 4 of the method. In this case, a simple conclusion results: if the treatment effect estimate increased by 50% when confounding amplification is expected to increase by 50%, this suggests that the entire, apparent treatment effect estimate that was observed in Model 1 is due to confounding.

In this simple scenario (i.e., involving no Introduced Variable-outcome association), the only way for a genuine treatment effect to exist, if the genuine treatment effect and unmeasured confounding are in the same direction, is if the Model 2 treatment effect estimate increased by an amount less than the proportional confounding amplification occurring from Model 1 to Model 2. This is because it is only the confounding that amplifies, not the treatment effect, as the propensity score model’s R ^2^ increases. In a sense, the treatment effect provides a “kernel” of constant effect amidst the change (amplification) of confounding. In this case, the more the original (Model 1) treatment effect estimate reflects genuine treatment effect, the more refractory the treatment effect estimate should be to amplification in Model 2.

Alternatively, if a genuine treatment effect existed in the opposite direction of confounding, then the change in the Model 2 treatment effect estimate would have to be *greater* than the amount predicted by strictly applying the expected proportional confounding amplification to the Model 1 treatment effect. This is because more confounding would be necessary than simply that needed to account for the difference between the treatment effect estimate and a null association: additional confounding would be required to also account for the “distance’ between the genuine treatment effect (in the opposite direction) and the null value. As a result, this additional confounding beyond that required to explain simply the entire treatment effect estimate (compared to the null) would lead to a change greater than predicted if the treatment effect estimate represented only the effect of confounding. In both these cases, the presence of a genuine treatment effect means that a change would be observed that was *different* (either greater or lesser) from that expected from the simple multiplication of the Model 1 treatment effect estimate by the amount of increased confounding amplification.

The second Hypothetical Example makes this clearer. Assume an identical scenario to the first example above, with only one difference: the Model 2 treatment effect estimate remains completely unchanged at RR 1.10. In this case the same 50% increase in confounding amplification between the two models produced a complete lack of a difference in the treatment effect estimates, implying essentially no residual confounding exists in Model 1. Furthermore, if residual confounding *is in the same direction* as the genuine treatment effect, the only way (absent an effect of random variability) that the Model 1 estimate can reflect a genuine treatment effect is if the Model 2 RR ends up between 1.10 and 1.14. A Model 2 estimate of RR = 1.15 would imply essentially no genuine treatment effect (Hypothetical Example 1), while an RR > 1.15 would imply that the treatment effect and residual confounding are in opposite directions (and that some degree of a genuine, protective treatment effect exists) ( Endnote D).

Hypothetical Example 3 examines a simplified version of the example provided in the Appendix Table. (The more complex version is discussed as Hypothetical Example 4). The simplification is to assume no association between the Introduced Variable and outcome. Assume Model 1 has a treatment effect estimate of RR = 1.265 (beta coefficient = 0.235072) and an R ^2^, in terms of prediction of exposure, of 0.25. Assume Model 2 has a treatment effect estimate of RR = 1.2985 (a beta coefficient = 0.2612) and an R ^2^ of 0.5. (This again would produce 50% confounding amplification). If the Introduced Variable has no association with outcome, we can immediately determine, by mere inspection, that confounding is relatively modest and the effect estimate of 1.265 primarily represents genuine treatment effect. The reason is that little change occurs in the treatment effect estimate. Certainly the increase in the treatment effect estimate does not come close to the value of RR = 1.422 that would be expected if the entire original RR = 1.265 was due to confounding (beta coefficient 0.235072 × 1.5 = 0.352608, which exponentiated equals 1.422). In fact, the difference in beta coefficients between Model 1 and Model 2 is just +0.0261. This means that, if 50% confounding amplification increases beta by 0.0261, then total confounding in Model 1 = 0.0261 / (1 - 0.75) / (1 - 0.5) = 0.0522, and thus the genuine treatment effect is beta = 0.2351 - 0.0522, or 0.1829, or RR = 1.20. This demonstrates that if no Introduced Variable-outcome association is present, then a treatment effect estimate that is generally refractory to the addition of the Introduced Variable(s) suggests that most of the effect estimate is genuine treatment effect.

The three examples above help demonstrate the important need to be able to accurately detect small differences in the treatment effect estimate between Model 2 and Model 1. In addition, the differences that are detected need to be due to confounding amplification, rather than due to other differences ( Appendix 2) or random variation. The next example illustrates the importance of accurately detecting and correcting for any Introduced Variable-outcome relationship.

Hypothetical Example number 4 illustrates the somewhat more complex, but still relatively straightforward, calculations required when the Introduced Variable does have an association with outcome. The full calculations have been described in the Methods section, with further detail provided in the Appendix Table. Let us return to Hypothetical Example 3 but assume that the Introduced Variable has an association with outcome of beta = 0.04879 [RR = 1.05]. We also need to know how the degree of imbalance in this variable initially between the treatment groups in Model 1 (in this example, there is an 80% [treatment group] to 20% [comparison group] imbalance), and how much more closely into balance it becomes in Model 2 (in this example, a 52% to 48% difference). Immediately, we can appreciate that the quantity of unmeasured confounding in the treatment effect estimate is considerably larger than in Hypothetical Example 3, for one simple reason: ordinarily, if we markedly reduce the imbalance in a variable that is more prevalent in the treatment group (as in this case) and which biases in the same direction as the treatment effect estimate, we would expect to see a *decrease* in the treatment effect estimate, not an *increase*. (The treatment effect beta coefficient increases by +0.0261). The fact that an increase is observed must mean that considerable additional confounding exists, separate from the effects of the Introduced Variable. This additional confounding must be large enough so that its relatively modest amplification (50%) is more than sufficient to overcome the effect of the increased balance in the Introduced Variable. Using the Bross equation allows us to quantify the expected effect of the increased balance of the Introduced Variable. This change in balance would be estimated to *decrease* the treatment effect estimate by beta = 0.0273. So, to determine the actual quantitative amount of amplified confounding, we subtract this decrease in treatment effect estimate from the change in the treatment effect estimate that was observed. Thus the change in the treatment effect estimate due to confounding amplification is beta = 0.0534 (i.e., 0.0261 minus -0.0273). Dividing this quantity by the predicted amplification of 50%, as determined by the calculation ((1 / (1 - 0.5)) / (1 / (1 - 0.25))) - 1 = 0.5, gives an estimate of total Model 1 residual confounding attributable to the amplifiable fraction (i.e., the total residual confounding except that contributed by the Introduced Variable and its correlates) of beta = 0.0534 / 0.5, or 0.1068.

We now must subtract this value, plus the confounding attributable to the Introduced Variable, from the Model 1 treatment effect estimate. At this point, the Bross equation uses the difference between an 80%/20% imbalance and the 50%/50% balance that would be observed if there was no confounding in Model 1 due to the Introduced Variable. This produces a slightly larger number (beta = 0.0293) than in the previous application of the Bross equation (beta = 0.0273), which estimated confounding resulting from the difference between the initial 80%/20% imbalance and the 52%/48% balance observed after propensity score balancing. Adding this beta = 0.0293 quantity to our estimate of the confounding attributable to the amplifiable fraction means that we estimate that Model 1 contained residual confounding of beta = 0.1068 + 0.0293, or 0.1361. Since the treatment effect estimate is RR = 1.265 (beta = 0.2351), this means the genuine treatment effect estimate is beta = 0.2351 - 0.1361, or beta = 0.09903 [i.e., RR = 1.10]). That is, the findings imply that more than half of the original, sizeable “treatment effect estimate” (beta = 0.2351; RR = 1.265) was attributable to residual confounding. (Please see the Appendix Table for complete calculations).

Put in the form of the Summary Equation, the following calculation of the unconfounded treatment effect estimate would result:

0.2351 - ((0.0261 - -0.273) / (((1 / (1 - 0.5)) / (1 / (1 - 0.25))) - 1)) - 0.293 = 0.09903

Thus, despite the fact that the treatment effect estimates for Model 1 and Model 2 are both confounded by an unknown amount of unmeasured confounding, it is possible, in principle, to derive an estimate of an unconfounded treatment effect. This estimate is possible because knowledge of the quantitative change between these two treatment estimates and the estimated proportional confounding amplification underlying this change allows, in a few steps, the derivation of an estimate of the unconfounded treatment effect.

## Results

### Partial application using published data

The example provided here from published data builds from a rare opportunity in the literature in which sufficient information has already been provided to partially apply the method. Thanks to their detailed reporting, Patrick *et al.*, ^10^ fortuitously present results that provide an opportunity to apply some aspects of the ACCE Methodology on real-world data. Obviously, their study was not constructed to illustrate the ACCE Method; therefore it is being used *post hoc* to explore the potential of the method. The full quantitative version of the ACCE Method cannot be applied for several reasons (discussed below). As a result, the data provided include several additional uncertainties beyond those that would accompany a deliberate implementation of the ACCE Method. However, by permitting the performance of even a partial version of the ACCE Method to be assessed, this study illustrates the value this method may have in serving as a probe to provide at least a qualitative sense of whether substantial residual confounding is likely, along with its likely direction.

Patrick *et al.*, ^10^ analyzed the associations between statins and both all-cause mortality and hip fracture using a number of propensity scores. For both of the outcomes, two of the propensity scores formed an important nested pair. One propensity score was nested within a slightly larger propensity score that only differed in a single added covariate (glaucoma diagnosis). Glaucoma diagnosis was considered to be a potential instrumental variable in these analyses. First, glaucoma diagnosis was strongly associated with treatment exposure (since the comparison group for both analyses consisted of individuals who used medications to treat glaucoma). Patients with a glaucoma diagnosis had an odds ratio for statin exposure of 0.07. That is, patients with glaucoma diagnosis had approximately 14:1 odds of being in the comparison group (the group receiving medications for glaucoma) than the statin treatment group. Second, it is plausible (although not certain) that glaucoma diagnosis lacks a substantial association with the outcomes of all-cause mortality and hip fracture, and thus may be functioning as an instrumental variable or near-instrumental variable. (Although not termed an “instrumental variable” originally ^10^, such a term was used for glaucoma diagnosis in these analyses in a subsequent manuscript describing these findings ^11^).

Several information gaps limit this example, however, making it not possible to derive quantitative estimates of unmeasured confounding. Patrick *et al.* ^10^ did not report R ^2^ since they used logistic propensity scores, but rather provided c statistics. The relationship of the c statistic to confounding amplification has not been examined, in contrast to the relationship between R ^2^ and confounding amplification. In addition, it is not possible to adjust for any association between the Introduced Variable (glaucoma diagnosis) with outcome, since the needed coefficient from a full multivariate regression containing all the propensity score covariates are not provided. The manuscript does note that the minimally-adjusted hazard ratio (HR) for glaucoma diagnosis (adjusted for age, age ^2^, and sex) is >1.175 or <1 / 1.175 for both outcomes. (The actual age and-sex-adjusted HR observed is HR ≈ 0.85 for both outcomes [Dr. Amanda Patrick, Personal Communication]). While the age- and sex-adjusted HR has some value, what is truly needed is the glaucoma diagnosis HR, adjusted for all the other propensity score covariates. (This would total 143 covariates for the mortality analysis and 120 covariates for the hip fracture analysis ^10^). This fully-adjusted HR would provide information about whether the glaucoma diagnosis HR would approximate a null value if all the other covariates were included. It is also not possible to determine if close similarity exists between the models in the balance achieved in the measured covariates and the intervention delivered (e.g., dose or duration) ( Appendix 2). Finally, the measure of treatment effect, the hazard ratio, may possibly complicate efforts to derive a quantitative estimate of confounding due to the noncollapsibility of the hazard ratio.

### Interpretation of the published results using a partial version of the ACCE Method

Despite such limitations, application of even this partial version of the ACCE Method appears to provide useful qualitative estimates of the residual confounding present in these analyses. Table 1A shows that in the all-cause mortality analyses the addition of the Introduced Variable (glaucoma diagnosis) moves the treatment effect estimate away from the null by a modest amount. This implies that the total residual confounding (including residual confounding from unmeasured factors) likely biases, but only very modestly, towards observing a larger effect size for statins than is genuinely present. This observation is consistent with randomized data ^12^. In contrast, Table 1B shows that addition of the same Introduced Variable in the hip fracture analysis changes the observed treatment effect HR from 0.76 to 0.69. This is a much more sizeable change, implying a larger quantity of underlying residual confounding biasing the estimate away from the null. If glaucoma diagnosis is in fact a near-instrumental variable, the results would suggest that the unconfounded treatment effect estimate is considerably closer (than HR = 0.76) to the null value suggested by randomized data ^13^.

**Table 1.** Examples of qualitative application of the ACCE Method (drawn from Reference 10).

Table 1A. Statin - mortality analysis			
**Model 1**			
**Exposure (Treatment) of Interest:** Receipt of Statin (vs. Glaucoma Medication) **Outcome:** All-Cause Mortality **Included Covariates:** 143 Variables with a +/- 20% association with All-Cause Mortality			
**c statistic**	**Treatment Effect** **Estimate** (Hazard Ratio)	**Expected Result** (from RCT meta-analyses)	**Likely Confounding and Treatment Effect** (based on comparison with RCT data)
0.82	HR = 0.84	HR = 0.85 or less (i.e., closer to null)	Away from null (treatment effect estimate overestimates statin protective effect), although confounding appears small to modest. Genuine treatment effect most likely closer to the null than HR = 0.84.
**Model 2** *(identical to Model 1 except for Addition of a Single “Introduced Variable”: Glaucoma Diagnosis)*			
**Exposure (Treatment) of Interest:** Receipt of Statin (vs. Glaucoma Medication) **Outcome:** All-Cause Mortality **Introduced Variable “Probe”:** Glaucoma Diagnosis, a variable with a strong association with exposure (approximately 14X more common in the comparison group [glaucoma medication users] than in the statin group) **Expected Association of Introduced Variable with Outcome:** Minimal **Included Covariates:** 143 Variables with a +/- 20% association with All-Cause Mortality, plus the “Introduced Variable”			
**c statistic**	**Treatment Effect** **Estimate** (Hazard Ratio)	**Size & Direction of Change** **of Treatment Effect** **Estimate**, compared to Model 1	**Likely Confounding and Treatment Effect** (based on ACCE Method)
0.90	HR = 0.82	Small (0.84 → 0.82) in direction away from null	Away from null (since amplifying confounding pushes treatment effect estimate in that direction), although confounding appears small to modest. Genuine treatment effect most likely closer to the null than HR = 0.84.
Table 1B. Statin - hip fracture analysis			
**Model 1**			
**Exposure (Treatment) of Interest:** Receipt of Statin (vs. Glaucoma Medication) **Outcome:** Hip Fracture **Included Covariates:** 120 Variables with a +/- 20% association with Hip Fracture			
**c statistic**	**Treatment Effect** **Estimate** (Hazard Ratio)	**Expected Result** (from RCT meta-analyses)	**Likely Confounding and Treatment Effect** (based on comparison with RCT data)
0.81	HR = 0.76	Approximately HR = 1.0	Away from null (treatment effect estimate overestimates statin protective effect), and confounding appears likely to be large. Genuine treatment effect most likely closer to the null than HR = 0.76, potentially substantially closer.
**Model 2** *(identical to Model 1 except for Addition of a Single “Introduced Variable”: Glaucoma Diagnosis)*			
**Exposure (Treatment) of Interest:** Receipt of Statin (vs. Glaucoma Medication) **Outcome:** Hip Fracture **Introduced Variable “Probe”:** Glaucoma Diagnosis, a variable with a strong association with exposure (approximately 14X more common in the comparison group [glaucoma medication users] than in the statin group) **Expected Association of Introduced Variable with Outcome:** Minimal **Included Covariates:** 120 Variables with a +/- 20% association with Hip Fracture, plus the “Introduced Variable”			
**c statistic**	**Treatment Effect** **Estimate** (Hazard Ratio)	**Size & Direction of Change of** **Treatment Effect Estimate**, compared to Model 1	**Likely Confounding and Treatment Effect** (based on ACCE Method)
0.89	HR = 0.69	Sizeable (0.76 → 0.69) in direction away from null	Away from null (since amplifying confounding pushes treatment effect estimate in that direction), and confounding might be large. Genuine treatment effect most likely closer to the null than HR = 0.76, potentially substantially closer.
**Comparison Between the Two Analyses**			
Use of the glaucoma diagnosis Introduced Variable “probe” suggests substantially more confounding in the hip fracture analysis than the all-cause mortality analysis. From a highly similar starting point (c = 0.81 or 0.82) and highly similar magnitude of c statistic change (0.08), the treatment effect estimate for the hip fracture analysis moved substantially further away from the null than for the all-cause mortality analysis. This matches what is suggested from RCT data.			
**Additional Considerations:** 1) For these two examples there are randomized trial meta-analyses (extrinsic information) to separately inform judgments about likely confounding. This helps boost confidence in the ACCE Method in that it provides the same qualitative conclusions about likely confounding that reference to the RCT meta-analysis provides. However, the ACCE Method is likely to have its greatest value in circumstances in which such meta-analyses are lacking, since it permits evidence from the analysis itself to inform judgments about confounding. 2) Several elements are lacking that are necessary to derive a quantitative estimate of residual confounding. Missing data elements include knowledge of whether (and how) c statistics reliably index confounding amplification in a manner analogous to R ^2^ values, and a regression coefficient for glaucoma diagnosis that includes all the covariates in the propensity score. What is known is that in age and sex-adjusted analyses, the association of glaucoma diagnosis with both outcomes (as measured by the hazard ratio) was extremely similar (HR = 0.85) (Dr. Amanda Patrick, personal communication). However, whether this similarity between the analyses in the Introduced Variable-outcome association persists in the full propensity score analyses is uncertain (since the two propensity score analyses contain at least some different covariates). Nor is it known if the glaucoma diagnosis-outcome associations in the fully multivariate regression are close to HR = 1.0 (which is possible, but far from certain).			
**Notes:** Data taken from Reference 10. Specifically, data for each “Model 1” presented here was taken from Table 2 of Reference 10 (the Outcome +/- 20% model, or the 6th model listed for both the all-cause mortality and the hip fracture outcomes). Data for each “Model 2” presented here was taken from information provided in the text of Reference 10 (page 554). HR = hazard ratio			

Even if glaucoma diagnosis is not functioning as a near-instrumental variable, as long as the fully-multivariate regression coefficients for glaucoma diagnosis for each outcome are generally similar (and the age- and sex-adjusted hazard ratios for glaucoma diagnosis presented previously are highly similar), the similar change in c statistics observed would suggest the presence of considerably more residual confounding in the hip fracture analysis than the all-cause mortality analysis ( Endnote E). This is a conclusion independently suggested by the randomized trial meta-analyses ^12, 13^ cited by the authors. Notably, the ACCE Method, even when applied in a very partial and qualitative form, suggests the same conclusion. In this fashion, the ACCE Method may prove useful for estimating at least the likely general size and direction of residual confounding in the many circumstances where substantial randomized trial data does not exist. This capacity of the method to provide even a qualitative estimate of residual confounding may constitute an important analytic advance.

## Discussion

This paper presents a relatively straightforward method exploiting the phenomenon of confounding amplification to potentially obtain quantitative estimates of total residual confounding and unconfounded treatment effects. To my knowledge, it has not been previously recognized that the phenomenon of confounding amplification, if predictable (as suggested by both recent simulation ^5^ and theoretical work ^4^), provides a potential mechanism to estimate total residual confounding. The fundamental approach of deliberately introducing amplified confounding into an analysis to evaluate, qualitatively or quantitatively, the total residual confounding originally present appears to possess both clear logic and considerable promise.

Even if subsequent research determines that ACCE Method estimates are too imprecise to serve as useful quantitative estimates, this general approach may have considerable value as a semi-quantitative or qualitative “probe” for detecting the general size and direction of residual confounding. While important facets of the method are not yet fully resolved concerning its quantitative accuracy and optimum implementation (see below), further research is clearly indicated given the potential value of a new approach to removing confounding from nonrandomized treatment effect estimates. It is hoped that the description of the method provided here is sufficient to permit the larger research community to immediately begin participating in the validation and refinement of this novel approach.

### Considerations for validation and further research

This method will have its greatest value to the extent it succeeds in providing a useful quantification of residual confounding. Establishing such performance by the method will involve more detailed and precise examination of both simulated and real-world data, and almost certainly will involve the contributions of multiple research teams. Useful avenues for validation research include (in anticipated priority order):

1) **Determining the predictability of the relationship between the proportional amount of confounding amplification and measures of exposure prediction or change in internal markers.** The predictability of the proportional amount confounding amplification is the linchpin of this proposed method. While this predictability is suggested by two publications ^4, 5^, ways can be envisioned in which this predictability might break down in real-world datasets ( Appendix 3.1, Appendix 3.2 and Appendix 3.3).

2) **Establishing that multivariate regressions can be used to accurately estimate the contribution of the Introduced Variable and its correlate(s) to both the original confounding and change in confounding between models.** This is discussed extensively in Appendix 3.2 and Appendix 4.

3) **Determining whether sufficiently precise results can be routinely obtained from the ACCE Method despite random variability in the treatment effect estimates**. Some recent studies do suggest that quite subtle changes in relative risk or hazard ratio resulting from the application of slightly different propensity score models can be detected ^9, 10^.

4) **Developing methodology to develop confidence limits around the ACCE Method’s final treatment effect estimate.** An obvious need for such methods exists. The procedure of bootstrapping would be one candidate approach.

5) **Identifying approaches to, or circumstances that would, ensure other differences between the two models (e.g., in the balance achieved for included confounders, in the patient sample, and in the intervention received) are minimized.** Whether these differences (discussed in Appendix 2) would create substantial error is uncertain.

An important need also exists to determine whether a set of Introduced Variables can be used, as appears possible ( Appendix 6), if a single Introduced Variable does not produce sufficient confounding amplification. Indeed, part of the imperative for research on the ACCE Method stems from the possibility that the method may have unusually broad flexibility by: 1) permitting estimation using variables with a substantial association with exposure but also having an independent associations with outcome; 2) permitting a set of variables to be used to predict exposure for the purposes of the method; and 3) possibly also functioning to permit estimates of unmeasured confounding *after* treatment initiation ( Appendix 2.3b).

Simulation studies will almost certainly be the most immediate approach to addressing these research needs and evaluating the performance of this method in general. (These studies have the advantage that given that the genuine treatment-outcome association and the amount of unmeasured confounding is able to be precisely specified by the investigator). Such simulations might build upon the recent simulation study reporting predictable confounding amplification within the lower range of R ^2 5^, and others that have considered the impacts of unmeasured confounding ^14, 15^. Simulations might start with a simple 4- or 5-variable scenario: the treatment, a measured confounder, the Introduced Variable, and one or two unmeasured confounders. The simulations might start by testing the accuracy of the treatment effect estimates achieved when the Introduced Variable does or does not have an association without outcome, and expand to examine whether, and how much, the performance of the method suffers when varying strengths of correlation exist between the Introduced Variable and one or more unmeasured confounders.

Real-world studies will also be needed to help resolve whether the ACCE Method, when applied to complex and often highly-correlated real-world data, succeeds in making results from nonrandomized studies better parallel results from randomized trials ^16, 17^ ( Appendix 3.2 and Appendix 7).

### Potential application of the method to comparative effectiveness and surveillance research

Even if this method ultimately does not demonstrate strong quantitative precision, the potential qualitative estimates of this method may prove to have some benefit for nonrandomized comparative effectiveness research in general, especially for studies in which substantial residual or unmeasured confounding is expected. For example, many studies of mental health and/or behavioral interventions might be expected to have substantial unmeasured confounding. Important elements of the conversation between patient and provider that inform judgments of the severity of the patient’s condition and help influence treatment decisions may often go unrecorded in administrative data or even in the patient’s chart, and thus be unmeasured.

Another notable use would be to enhance medication surveillance efforts. By providing even an approximate sense of whether substantial unmeasured confounding is likely to be present, the ACCE Method could help more accurately indicate which prominent “signals” (either in effectiveness or safety) observed during the screening of large datasets appear to be less confounded (and thus should be a priority for additional investigation).

## Conclusions

This paper has outlined a relatively straightforward yet novel method to potentially obtain a quantitative estimate of total residual confounding. This total residual confounding estimate then allows, in principle, for an estimate of the unconfounded treatment effect to be calculated. This paper has described the two overarching components of the method and described the specific individual steps and substeps necessary for its implementation. This paper has also offered a preliminary examination of the performance of a simple, partial version of this method on published data, and outlined research needs for refinement and validation of this method. Given the importance of identifying methods that may help remove confounding from nonrandomized treatment effect estimates, further investigation of this method by multiple research groups is clearly warranted. Even if the ACCE Method is eventually shown to have limitations or evolves from the form proposed here, the method’s general approach of deliberately amplifying confounding to permit estimation of the residual confounding originally present may have enduring analytic value. The ACCE Method and its underlying logic therefore have the potential to constitute a substantial advance for nonrandomized intervention research, and follow-up research should be rapidly conducted.

### ENDNOTES

A. In actual application, these calculations need to account for any association between the Introduced Variable(s) and outcome if present. This adjustment is not included in this very simple example, but is likely needed in most implementations of the method (and is discussed in Steps 3 and 4).
B. These regressions could be performed either within treatment arms or across both treatment arms while including an indicator for treatment arm, as well as a covariate(s) for treatment arm-Introduced Variable interaction(s). My expectation is that regressions within each treatment arm may be more useful, given the correlation between the Introduced Variable and treatment, although this approach raises the question about how to best combine the two within-treatment arm coefficients (e.g., an average, weighted by the number of patients in each treatment arm).
C. This example and the subsequent examples use a linear propensity score model and a linear (risk ratio) outcome model. This is because: 1) the existing simulation demonstrating proportional confounding amplification is for a linear propensity score model, and 2) it is conceivable (but not certain) that the noncollapsibility of logistic outcome models might interfere with the accuracy of the subtraction of the Model 1 treatment effect estimate from the Model 2 treatment effect estimate.
D. The only way to produce such a large quantitative change in the treatment effect estimate (i.e., RR > 1.15) with 50% confounding amplification and starting from an RR of 1.10 would be for the unmeasured confounding to be so substantial as to exceed the entire Model 1 treatment effect estimate. Subtracting this estimated amount of confounding from the Model 1 treatment effect estimate would therefore produce an estimate of the genuine treatment effect that was below the null (ignoring, once again, random variability and making the important assumption of no association between the Introduced Variable and outcome).
E. In fact, the change in c statistic is *highly* similar (all-cause mortality: Model 1 c = 0.82, Model 2 c = 0.90; hip fracture: Model 1 c = 0.81, Model 2 c = 0.89). Thus, even though it is not clear whether the c statistic in general can serve as even an approximate index of confounding amplification, the c statistics in this case are so similar as to suggest similar proportional confounding amplification is likely. The much greater change in the treatment effect estimate observed for the hip fracture analysis implies this analysis contains greater residual confounding biasing in the direction of a protective effect, if the assumption made, that the fully-adjusted glaucoma diagnosis HR is similar in the two analyses, is valid.
F. At the most fundamental level, the ACCE Method can be conceptualized as determining the unconfounded treatment effect estimate by subtracting from the Model 1 Treatment Effect Estimate both a) the estimate of confounding in Model 1 for the “amplifiable fraction” of Model 1 confounding (that is, the confounding from all the confounders *except* the Introduced Variable(s) and, to some degree, its correlates), and b) subtracting the confounding due to the Introduced Variable, and, to some degree, its correlates. The terms described in “a)” and “b)” are intended to be complements of each other, in that together they are intended to encompass between them all the (baseline) confounding present in Model 1. Thus, what remains when they are removed is the unconfounded treatment effect estimate for Model 1. (The most rigorous, but much more labor-intensive, estimate of Model 1 residual confounding would also include the confounding contributed by the residual imbalance of each of the propensity score covariates and, potentially, correlates of these covariates, as well as the change in the balance of these propensity score covariates occurring between Model 1 and Model 2. For simplicity, those terms are not considered here, but are discussed in Appendix 2.1, Appendix 3.2a, and Appendix Figure 1c).
  The quantities that we can estimate by relatively conventional analysis of the data (i.e., in a sense, the “known quantities”) are the Model 1 treatment effect estimate and the estimated confounding attributable to the Introduced Variable(s) and its correlates (by using multivariate regression coefficients and the Bross equation). Therefore, the *only term for which we are lacking an estimate* is the confounding due to the “amplifiable fraction” of Model 1 confounding. Furthermore, we can obtain important information that bears upon the confounding attributable to the “amplifiable fraction” of Model 1 confounding. This information consists of the Model 2 treatment effect estimate, and the proportional amount of confounding amplification expected, which recent simulation and theoretical work suggests can be predicted for linear propensity score models from the model R ^2^ (for predicting exposure). More precisely, the proportional confounding amplification is estimated from (1 / (1 - R ^2^)).
  The most fundamental contribution of the ACCE Method is to call to attention to the fact that, with this readily available information, the final term needed (the Model 1 confounding attributable to the “amplifiable fraction”) should be able to be estimated. While the accuracy of this estimate has yet to be determined, the ability to come up with even an approximate estimate of the aggregate effect of all remaining residual confounding is noteworthy. In the manuscript and Appendices, the confounding in Model 1 attributable to the “amplifiable fraction” is represented by the following term:
  $$\left(\frac{{(\text{TEE}}_{\text{M2}}-{\text{TEE}}_{\text{M1}})-{\text{Conf}}_{{\text{IntV}}_{\Delta \text{(M2}-\text{M1)}}}}{\left(\left(\frac{\frac{1}{\left(1-R_{\text{M2}}^{\text{2}}\right)}}{\frac{1}{\left(1-R_{\text{M1}}^{\text{2}}\right)}}\right)-1\right)}\right)$$
  where TEE _M2_ equals the Model 2 Treatment Effect Estimate, TEE _M1_ equals the Model 1 Treatment Effect Estimate, Conf _IntV _Δ(M2-M1)__ equals the confounding associated with the change in the balance between the treatment groups of the Introduced Variable(s) and its correlates in Model 2 compared to Model 1, and *R* ^2^_M1_ and *R* ^2^_M2_ equal the *R* ^2^ values for propensity score Model 1 and Model 2, respectively.
  To derive this term mathematically, we can proceed with the following reasoning. Consider the simplest, “ideal” case in which no changes occur between Model 1 and Model 2 except confounding amplification. For example, that would mean that there are no differences between Model 1 and Model 2 in the balance in measured confounders or in the ”dose” of intervention received (although it is important to recognize, as discussed in Appendix 2, that means exist to address differences in either of these characteristics). Then the difference in the Model 2 treatment effect and the Model 1 treatment effect, once the differences attributable to the change in balance of the Introduced Variable are taken into account (i.e., Conf _IntV _Δ(M2-M1)__) should reflect amplification of the Model 1 confounding that was not attributable to the Introduced Variable(s) and, to some degree, its unmeasured or nonincluded correlates. For this case, let us assume an Introduced Variable or Variables that is not correlated with other unmeasured or nonincluded confounders. To represent the difference in treatment effect estimates independent of the contribution from the change in balance of the Introduced Variable as it is added to generate Model 2, we can use the term:
  (TEE _M2_ - TEE _M1_) - Conf _IntV _Δ(M2-M1)__
  where TEE _M2_, TEE _M1_, and Conf _IntV _Δ(M2-M1)__ are as defined above.
  For simplicity for the next few steps, let us substitute a single term for this quantity, “AdjΔ _TEE_”, with “Adj” referring to “Adjusted”, that is, this change in Treatment Effect Estimates between Model 2 and Model 1 has been adjusted to reflect the contribution from the change in balance of the Introduced Variable. The “AdjΔ _TEE_” terminology matches that used in Steps 3d and 4a in Appendix Table 1. That is:
  (TEE _M2_ - TEE _M1_) - Conf _IntV _Δ(M2-M1)__ = Adj _ΔTEE_
  Since we are examining conditions where this AdjΔ _TEE_ reflects only the amplification of the amplifiable fraction of Model 1 confounding, this term can be set equal to this amplification. This amplification can be represented algebraically, using the 1 / (1 - R ^2^) relationships, in a simple form, as follows, starting with the Model 1 Treatment Effect Estimate:
  Model 1 Treatment Effect Estimate = *x* + *y* + *k*
  where *x* equals confounding from the amplifiable fraction in Model 1, *y* equals the confounding due to the Introduced Variable and, to some degree, any nonincluded correlated confounders, and *k* equals the constant that is of crucial interest (the unconfounded treatment effect estimate). With this notation, Model 2’s Treatment Effect Estimate can be represented as:
  $$\mathrm{Model}2\mathrm{Treatment}\mathrm{Effect}\mathrm{Estimate}=\left(\left(\frac{\frac{1}{\left(1-R_{\text{M}2}^{2}\right)}}{\frac{1}{\left(1-R_{\text{M}1}^{2}\right)}}\right)x\right)+y+k$$
  In this equation, the term $\left(\frac{\frac{1}{\left(1-R_{\text{M}2}^{2}\right)}}{\frac{1}{\left(1-R_{\text{M}1}^{2}\right)}}\right)$ represents the proportional amount of additional confounding in Model 2 compared to Model 1. Confounding amplification in Model 2 cannot be estimated simply by $\left(\frac{1}{\left(1-R_{\text{M}2}^{2}\right)}\right)$ because that would represent the confounding amplification relative to a model with an R ^2^ of 0.0. Instead, the proportional amount of confounding amplification in Model 2 relative to Model 1 needs to be determined, and for this reason the $\left(\frac{1}{\left(1-R_{\text{M}2}^{2}\right)}\right)$ term is divided by $\left(\frac{1}{\left(1-R_{\text{M}1}^{2}\right)}\right)$, creating the term $\left(\frac{\frac{1}{\left(1-R_{\text{M}2}^{2}\right)}}{\frac{1}{\left(1-R_{\text{M}1}^{2}\right)}}\right)$.
  Given these terms for the Model 1 and Model 2 Treatment Effect Estimates, then AdjΔ _TEE,_ the Model 2 – Model 1 change in the Treatment Effect Estimate, adjusted to reflect only amplification of confounding (that is, not the change attributable to insertion of the Introduced Variable(s) “probe” needed to generate confounding), can be expressed as:
  $$\text{Adj}\Delta_{\text{TEE}}\;\text{=}\;\left(\left(\left(\frac{\frac{1}{\left(1-R_{\text{M}2}^{2}\right)}}{\frac{1}{\left(1-R_{\text{M}1}^{2}\right)}}\right)x\right)+y+k\right)-(x+y+k)$$
  Focusing on the right side of the equation, the *y* and *k* terms cancel out and can be removed: + *y* + *k* - ( + *y* + *k*) = 0. This makes intuitive sense. Regarding *y*, the component of y that is unchanging (i.e., in common between Model 1 and Model 2) cancels out and is not part of the difference between TEE _M2_ and TEE _M1_. Regarding *k*, the genuine underlying treatment effect estimate has not changed between Model 1 and Model 2, assuming that we can keep elements such as balance in the propensity score covariates and/or dose received the same or, for practical purposes, extremely similar between the models. If the genuine treatment effect is not varying between models, then it is not making a contribution to the change in the treatment effect estimates between Model 2 and Model 1. (Alternatively, in the case of the balance of measured confounders included as covariates in the propensity score, the effect of changes in balance of those confounders could be estimated by the Bross equation [ Appendix 2.1 and Appendix Figure 1c]).
  This gives the equation:
  $$\text{Adj}\Delta_{\text{TEE}}\;\text{=}\;\left(\left(\frac{\frac{1}{\left(1-R_{\text{M}2}^{2}\right)}}{\frac{1}{\left(1-R_{\text{M}1}^{2}\right)}}\right)x\right)-x$$
  Next, we can factor out *x* to produce the following term.
  $$\text{Adj}\Delta_{\text{TEE}}\;\text{=}\;\left(\left(\frac{\frac{1}{\left(1-R_{\text{M}2}^{2}\right)}}{\frac{1}{\left(1-R_{\text{M}1}^{2}\right)}}\right)-1\right)x$$
  Solving for *x* yields:
  $$\left(\frac{\text{Adj}\Delta_{\text{TEE}}}{\left(\left(\frac{\frac{1}{\left(1-R_{\text{M}2}^{2}\right)}}{\frac{1}{\left(1-R_{\text{M}1}^{2}\right)}}\right)-1\right)}\right)=x$$
  Substituting back in the ((TEE _M2_ - TEE _M1_) - Conf _IntV _Δ(M2-M1)__) term for AdjΔ _TEE_ yields the equation for the original confounding (in Model 1) attributable to the amplifiable fraction of confounding, *x*:
  $$\left(\frac{({\text{TEE}}_{\text{M2}}-{\text{TEE}}_{\text{M1}})-{\text{Conf}}_{{\text{IntV}}_{\Delta \text{(M2}-\text{M1)}}}}{\left(\left(\frac{\frac{1}{\left(1-R_{\text{M2}}^{\text{2}}\right)}}{\frac{1}{\left(1-R_{\text{M1}}^{\text{2}}\right)}}\right)-1\right)}\right)=x$$
  Of note, the left hand side of the equation consists *entirely of terms that can be readily estimated* from data. This gives us the final quantity needed to estimate an unconfounded treatment effect estimate. Therefore, if we subtract this term plus an estimate of the Model 1 Confounding due to the Introduced Variable (s) and, to some degree, its correlates, from the Model 1 Treatment Effect Estimate, we should obtain an estimate of an unconfounded treatment effect:
  $$TEE_{M\text{1}}-\left(\frac{{(\text{TEE}}_{\text{M2}}-{\text{TEE}}_{\text{M1}})-{\text{Conf}}_{{\text{IntV}}_{\Delta \text{(M2}-\text{M1)}}}}{\left(\left(\frac{\frac{1}{\left(1-R_{\text{M2}}^{\text{2}}\right)}}{\frac{1}{\left(1-R_{\text{M1}}^{\text{2}}\right)}}\right)-1\right)}\right)-{\text{Conf}}_{{\text{IntV}}_{\text{M1}}}=\begin{array}{c} Unconfounded\\ TEE \end{array}$$
  Quantities are as defined previously, with the additions of Conf _IntV _M1__, which represents the confounding association with the Introduced Variable and, to some degree, its correlates, given the initial imbalance of the Introduced Variable observed in Model 1, and *Unconfounded TEE,* which represents the unconfounded treatment effect estimate that thereby results. As a point of clarity, no subscript is given to the *Unconfounded TEE* term to designate it as being the *Unconfounded TEE* for Model 1, even though the equation develops its *Unconfounded TEE* by subtracting the two components of Model 1 confounding from the observed Model 1 treatment effect estimate. No subscript is used because a key assumption is that the underlying treatment effect estimate for both models is the same.
  As pointed out in the manuscript, Appendix 3.2, and Appendix 4, the ability of the Introduced Variable(s) coefficient(s) to capture the contribution of confounding from the unmeasured or nonincluded confounders that are correlated with the Introduced Variable is a major source of uncertainty for the method. While it may (or may not) be determined that often this is not a major practical concern, or that Introduced Variables can be identified that largely lack any significant correlation with unmeasured confounding ( Appendix 4), further research is clearly needed. It should be somewhat straightforward to use simulated datasets to make at least an initial inquiry into the impact of unmeasured confounding correlated with the Introduced Variable on the unconfounded treatment effect estimate that this method produces. Another question concerns whether real-world constraints exist that may limit confounding amplification. A more complete list of potential uncertainties is provided in Appendix 5, Appendix Figure 1b.

## Figures and Tables

**Appendix Figure 1a.**
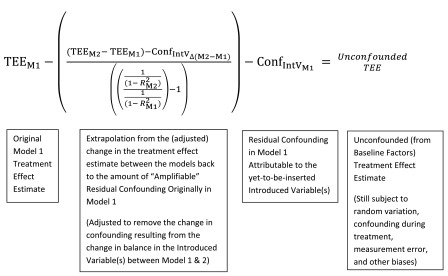
Components of the ACCE Method Summary Equation.

**Appendix Figure 1b.**
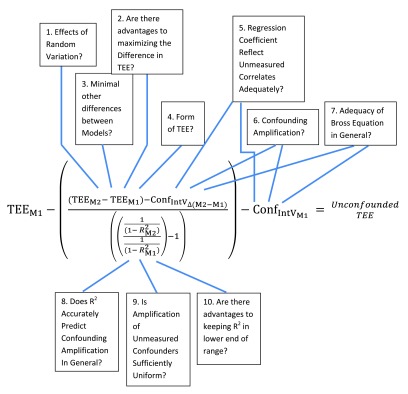
ACCE Method Summary Equation Annotated with Key Uncertainties. Abbreviations for Appendix Figure 1a and Appendix Figure 1b: TEE = Observed Treatment Effect Estimate, observed from outcome models when the particular propensity score model noted by the subscript is used to stratify or match the samples being compared. Unconfounded TEE = The Estimate of the Treatment Effect Unconfounded by Baseline Confounding through operation of the ACCE Method. M1 = Model 1. M2 = Model 2. IntV = Introduced Variable. Conf _IntV _Δ(M2-M1)__ = the change in confounding between Model 2 and Model 1 attributable to the change (i.e., expected decrease) in the imbalance of the Introduced Variable between Model 1 and Model 2. This is calculated through knowledge of the imbalance of the variable in both models and use of the Bross equation. Specifically, Conf _IntV _M1__ = Confounding attributable to the Introduced Variable in Model 1 1 - R ^2^ = 1 minus the R ^2^ observed for the propensity score model, i.e., the R ^2^ for the prediction of treatment exposure. The reciprocal of (1 - R ^2^), i.e. 1 / (1 - R ^2^) is the predictor of the amplification of confounding occurring during stratification or matching using that propensity score model. The subscript M1 and M2 denote the R ^2^ for the propensity score Model 1 and propensity score Model 2.

**Appendix Figure 1c.**
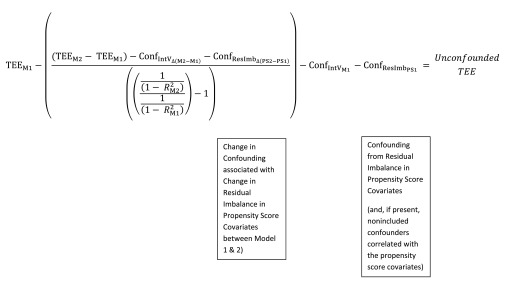
ACCE Method Summary Equation including additional elements relating to residual confounding attributable to the residual imbalance in the covariates included in both propensity scores ( Appendix 2.1 and Appendix 3.2a). Abbreviations for Appendix Figure 1c. Same as for Appendix Figure 1a and Appendix Figure 1b (above), plus: Conf _ResImb _Δ(PS2-PS1)__ = Confounding associated with the change (if any) in the residual imbalance between treatment groups observed in propensity score covariates in Model 1 versus Model 2. This can be estimated through inserting multivariate regression coefficients for each covariate into the Bross equation, incorporating information about the change in balance of the propensity score covariates in Model 1 and Model 2. I use the more general term “associated with”, rather than “attributable to”, to reflect the fact that changes in the balance of these included covariates would be expected to also result in change in the balance of the “included fraction” of nonincluded confounders that are correlated with these covariates. How well the Bross equation adjusts for these changes is uncertain. Conf _ResImb _PS1__ = Confounding associated with the residual imbalance in propensity score covariates in Model 1, and, if present, the correlated fraction of nonincluded confounders correlated with the included propensity score covariates. The effect of the residual imbalance in propensity score covariates in Model 1 upon residual confounding may be able to be estimated through the use of the Bross equation. The ability for this estimate to include the impact upon residual confounding of any nonincluded correlates of these propensity score covariates, however, it is less certain.

**Appendix Table 1.** Step-by-step application of the ACCE Method (hypothetical example). *Scenario:* A genuine treatment effect risk ratio (RR) = 1.10 exists for the investigated intervention (e.g., medication, surgery, psychotherapy, etc.). This association, however, is concealed by a larger amount of residual confounding (RR = 1.15) in the initial model (Model 1). This leads to bias in the observed association between the intervention and outcome in Model 1 (treatment effect estimate of RR = 1.265). Model 1 has an R ^2^ of 0.25. Applying the ACCE Method, an additional variable or variable(s) is identified that is substantially associated with treatment. This identified variable has an association of RR = 1.05 with the outcome, and a 4:1 imbalance (80% versus 20%) between the treatment groups in Model 1. Upon introduction into Model 2, this imbalance changes to a 1.08:1 imbalance (52% versus 48%) once this variable is included in the propensity score and balanced through stratification or matching. Model 2 (which has an R ^2^ of 0.5) has a treatment effect estimate of RR = 1.2985.

Step	Description	Verbal and Symbolic Formula	Example
1a	Construct propensity score Model 1 (“M1”) and determine its prediction of exposure and derive a Treatment Effect Estimate when the propensity score is used to stratify or match the treatment groups	Model 1 Treatment Effect Estimate = TEE _M1_ Model 1 prediction of exposure (in this case, using the metric R ^2^) = R ^2^_M1_	TEE _M1_ = RR _M1_ = 1.265 *alternatively*: TEE _M1_ = β _M1_ = ln(1.265) = 0.2351 R ^2^_M1_ = 0.25
1b	Construct propensity score Model 2 (“M2”) and determine its prediction of exposure and derive a Treatment Effect Estimate when the propensity score is used to stratify or match the treatment groups	Model 2 Treatment Effect Estimate = TEE _M2_ Model 2 prediction of exposure (again using the metric R ^2^) = R ^2^_M2_	TEE _M2_ = RR _M2_ = 1.2985 *alternatively*: TEE _M2_ = β _M2_ = ln(1.2985) = 0.2612 R ^2^_M2_ = 0.5
2	Estimate Proportional Confounding Amplification (“CAmp _Prop_”) (between Model 1 and 2) (either through use of a Prediction of Exposure metric or an “Internal Marker”) ^a^	*For R ^2^, if both R ^2^ < 0.56, THEN:* Proportional Confounding Amplification between Models = Inverse of (Proportional Amplification in Model 2 divided by Proportional Amplification in Model 1) Using R ^2^, Proportional Amplification of each model is given by (1 / (1 - R ^2^)), so this would equal: (1 / (1 - R ^1^_M2_) / (1 / (1 - R ^2^_M1_)) = CAmp _Prop_^b^	CAmp _Prop_ = (1 / 0.5) / (1 / 0.75) = 1.5
**Step 3** This step adjusts the change in the observed treatment effect estimate between Model 2 and Model 1 by the contribution that is attributable to the increased balance in the Introduced Variable or variables that produced the confounding amplification. Performing this simple adjustment requires 4 substeps, and in the case of Step 3b, further substeps within that subset.			
3a	Determine if an association (“IntV: O”) exists between the Introduced Variable ^b^ (“IntV”) and the Outcome (“O”) by examining the association within the treatment arms for each Model ^c^	IntV:Outcome Association = IntV:O or, as mean value, “RRi:o” More specifically: IntV:O _Model 1Treatment Group 1_ = IntV:O _M1 Tx Grp 1_ IntV:O _Model 1Treatment Group 2_ = IntV:O _M1 Tx Grp 2_ AND IntV:O _Model 2Treatment Group 1_ = IntV:O _M2 Tx Grp 1_ IntV:O _Model 2Treatment Group 2_ = IntV:O _M2 Tx Grp 2_	IntV:O _M1 Tx Group 1_ = 1.045 (as RR) IntV:O _M1 Tx Group 2_ = 1.055 (as RR) Mean IntV:O _1_ = RR _i:oM1_ = 1.05 ^d^ IntV:O _M2 Tx Group 1_ = 1.046 (as RR) IntV:O _M2 Tx Group 2_ = 1.056 (as RR) Mean IntV:O _2_= RR _i:oM2_ = 1.051 ^d^
**Step 3b** Determine the degree to which change in the Treatment Effect estimate is due to increased balance in the Introduced Variable.			
3b1	Estimated the Confounding attributable to Original (Model 1) Imbalance in Introduced Variable (“CIntV _M1_”) This is done through use of the Bross equation. ^e^	p = probability (e.g., 80%) ln [(p _M1 Tx Grp 1_ * (RR _i:oM1_ - 1) + 1) / (p _M1 Tx Grp 2_ * (RR _i:oM1_ - 1) + 1)] ln [(p _M2 Tx Grp 1_ * (RR _i:oM2_ - 1) + 1) / (p _M2 Tx Grp 2_ * (RR _i:oM2_ - 1) + 1)]	CIntV _M1_ = ln[(0.8 * (1.05 - 1) + 1) / (0.2 * (1.05 - 1) + 1)] = 0.0293
3b2	Estimated Confounding attributable to the Subsequent (Model 2) Imbalance in Introduced Variable (“CIntV _M2_”)	ln [(p _M2 Tx Grp 1_ * (RR _i:oM2_ -1) + 1) / (p _M2TxGrp2_ * (RR _i:oM2_ - 1) + 1)]	CIntV _M2_ = ln[(0.52 * (1.051 - 1) + 1) / (0.48 * (1.051 - 1) + 1)] = 0.002
3b3	Determine the change in Treatment Effect Estimate observed between Model 1 and Model 2 that is attributable to the increased balance in the Introduced Variable (resulting from stratification or matching on the propensity score).	Change in Effect Estimate (Model 2 - Model 1) attributable to increased balance of Introduced Variable (“Δ _TEE(IntV)_”): CIntV _M2_-CIntV _M1_ = Δ _TEE(IntV)_	Δ _TEE(IntV)_ = 0.002 - 0.0293 = -0.0273
3c	Calculate the Change in the observed Treatment Effect Estimate (Model 2 versus Model 1)	Model 2 Effect Estimate - Model 1 Effect Estimate = Change in Effect Estimate (“Δ _TEE(CAmp)_”): TEE _M2_ - TEE _M1_ = Δ _TEE(CAmp)_	Δ _TEE(CAmp)_ = 0.2612 - 0.2351 = 0.0261
3d	Adjust the Change in the observed Treatment Effect Estimate from Model 1 to Model 2 by the amount of change accounted for by increased balance in the Introduced Variable	Change in Effect Estimate (Model 2 - Model 1) - Difference in Treatment Effect Estimate attributable to the Introduced Variable = Adjusted Treatment Effect Estimate Change (“AdjΔ _TEE_”): Δ _TEE(CAmp)_ - Δ _TEE(IntV)_ = AdjΔ _TEE_	AdjΔ _TEE_ = 0.0261 - (-0.0273) = 0.0534
**Step 4** Determine the Residual Confounding from the “Amplifiable Fraction” and the Introduced Variable, the sum of which provides an estimate of Total Residual Confounding, and Subtract this Sum from the Model 1 Treatment Effect Estimate to obtain an unconfounded treatment effect estimate. This starts by calculating the estimate of Residual Confounding due to the “Amplifiable Fraction,” through extrapolation by dividing the adjusted change in the treatment effect estimates by the proportional difference in confounding amplification expected between the two models.			
4a	Calculate estimate of Residual Confounding in Model 1 except for the Introduced Variable by dividing the adjusted change in Treatment Effect Estimate by the effect of the change in amount of confounding amplification between the models. That is, by the ratio of the confounding amplification expected in each model	Adjusted Treatment Effect Estimate Change / (Confounding Amplification - 1) = Residual Confounding _Model 1 except for IntV_ (“CRes _M1-IntV_”): AdjΔ _TEE_ / (CAmp _Prop_ - 1) = CRes _M1-IntV_	CRes _M1-IntV_ = 0.0534 / ((1 / (1 - R ^2^_M2_) / (1 / (1 - R ^2^_M1_)) - 1)
**Step 4b** Determine the Total Residual Confounding in Model 1 by taking the result of Step 4a, adding the original contribution of the Introduced Variable or variable(s), and then subtracting this sum from the Model 1 Treatment Effect Estimate to obtain an estimate of the unconfounded Treatment Effect Estimate (for Model 1).			
4b1	Derive an estimate of Total Residual Confounding in Model 1	Residual Confounding _Model 1except for IntV_ + IntV _M1_ Confounding = Total Residual Confounding _Model 1_ (“CTotRes _M1_”): CRes _M1-IntV_ + CIntV _M1_ = CTotRes _M1_	CRes _1_ = 0.1068 + 0.0293 = 0.1361 e ^0.1398^ = 1.15 RRTotal Unconfounded (Model1)= 1.15f
4b2	Derive an Estimate of the Unconfounded Treatment Effect	Model 1 Effect Estimate - Total Residual Confounding _Model 1_ = Unconfounded Treatment Effect Estimate (“TEE _UnC_”): EE _M1_ – CTotRes _M1_ = TEE _UnC_	TEE _UnC_ = 0.2351 - 0.1361 = 0.0990 e ^0.0953^ = 1.10 RRUnconfounded (Model 1)= 1.10f,g

## Appendices

Seven appendices are provided to more thoroughly outline key considerations involved in the implementing the method. These Appendices are intended to provide detailed information that some readers may find very useful, but that may not be of interest to some other readers. These Appendices can be read in their entirety, selectively, or not at all.

Appendix 1 starts with simple metaphors designed to make the basic concept of why increasing a bias (confounding) can be useful for understanding the bias, and transitions into a more complex metaphor to illustrate the logical basis of the key elements of the method. These elements include: 1) the step involving the division of the change of treatment effect estimates by expected confounding amplification to back-extrapolate the original contribution of the “amplifiable fraction” of residual confounding; and 2) the need to subtract confounding due to the Introduced Variable (or change in the Introduced Variable) from both the change in treatment effect estimates and the original Model 1 treatment effect estimate.

Appendices 2, Appendix 3, and Appendix 4 address important questions concerning the need to attend to details of the comparison between Model 1 and Model 2 in order to obtain as accurate an estimate of total residual confounding as possible. Appendix 5 present the Summary Equation of the ACCE Method three times, once labeling the constituent parts, once noting how the various terms of the equation relate to the key uncertainties discussed in the manuscript and in Appendices 2 – Appendix 4, and once providing a particularly rigorous version adding adjustments discussed in Appendix 2 and Appendix 3. Appendix 6 starts to consider how multiple Introduced Variables (i.e., introducing a set of variables, rather than a single variable) might be used. Appendix 7 offers a discussion of the some of the qualities that can be currently anticipated as important to consider in choosing Introduced Variables and implementing the method.

Some may view this level of detail concerning important aspects of the method as premature, since the method has not been thoroughly validated yet. I hope instead that this detailed description of the issues that I have been able to anticipate will both facilitate the proper validation and accelerate the sophisticated use of the method going forward. The details discussed below may not be highly important if the method is ultimately determined to only provide general, highly-approximate qualitative estimates of residual confounding. If, however, this method indeed appears to provide, at least in some circumstances, beneficial quantitative estimates of residual confounding, then the details discussed below and even further details yet to be identified may prove important to consider.

## Appendix 1. Simple metaphors to represent the overall strategy and logic of the method

The conceptual underpinnings of the proposed method described in the manuscript can be communicated through a variety of metaphors, starting with the very simple and progressing to the more complex. Appendix 1.1a and Appendix 1.1b provides some simple examples that might be seen as a starting point for a largely nonquantitative appreciation of the method’s approach. Appendix 1.2 provides a fuller and somewhat more complex, but still minimally mathematical, metaphor that is intended to communicate the logical basis behind each of the major steps of the method. Appendix 1.3 builds on that metaphor to explore some of the current uncertainties about the method.

### 1.1a. A simple example based on growth of “harmful” bacteria

To start at the simplest level, one point of confusion about the method might involve the question of why an investigator would deliberately introduce *additional* confounding bias into their analytic models. Why amplify confounding? One extremely simple metaphor is provided by the practice of culturing blood to identify pathogens. Bacteria that are obtained from a patient blood sample are then grown for a period of time in media, thus “amplifying them”. As the bacteria grow in number they become easier to study. Thus, we amplify something we don’t want (the bacteria) to learn more about them and enable us to better eliminate them. The same is true, in a general sense, of confounding in the ACCE Method.

This metaphor can be taken further without adding too much complexity. Suppose one wanted a sense of which and/or how many bacteria were present in a sample that contained both harmful bacteria and beneficial white blood cells. By placing the sample into nutrients where only the bacteria would grow and increase in number, rather than the white blood cells, it should be possibly to get a sense of whether, and how many harmful bacteria are present. In this example, the harmful bacteria would represent the harmful confounding. We want to get a sense of how numerous these bacteria are within the sample. The white blood cells would represent the treatment effect estimate. When Model 2 is constructed, the intent is to amplify the “harmful” bias from confounding while keeping the “helpful” treatment effect estimate as constant as possible.

Finally, to go even further with this metaphor, if we knew the ultimate number of bacteria present, the typical dividing time for the bacteria growing in this set of nutrients (that is, the time it takes them on average to double their number), and we knew how long we grew the sample in the nutrients, we should be able to extrapolate backwards to the number of bacteria originally present. In this refinement, knowledge of how rapidly the bacteria divide in that particular set of nutrients would represent the knowledge of the proportional amount of confounding amplification expected to occur between Model 1 and Model 2.

### 1.1b. A simple metaphor based on microphones (sound amplification systems)

To perhaps allow some additional numerical “sense” of the workings of the method, consider the case of turning up the volume on a television, radio, or cell phone. If one knew that turning up the volume a certain amount doubled the volume (if, for instance, the volume settings were genuinely proportional, so that turning up the volume from “20” to “40” doubled the sound produced), and one knows the actual volume obtained after this doubling occurred, it should be both possible and simple to extrapolate back to determine what the original volume was. That is, it would not be necessary to know the original volume if you knew these other two quantities (the final volume, and the proportion by which the volume changed). In nonrandomized studies, one never knows exactly the “volume”, or amount, of total confounding, so an additional wrinkle that the ACCE Method uses is to measure the *change* in the overall effect estimate that occurs between two models. (Furthermore, this change is measured when circumstances have been deliberately constructed so that the models differ to the minimum extent feasible, other than the differences resulting from the amplification of confounding).

To make our example even more comparable, consider the scenario in which one knew that a particular type of microphone would double (i.e., “amplify”) the static, or white noise, in whatever sound is being broadcast. If one knew the original *total, or overall,* volume (on a linear scale) was “90 units”, and when we changed to that type of microphone the total volume increased to 93 units, then we would know that the static made up 3 units of the original sound (since doubling it added 3 units). This would mean that the volume devoid of any static would be 87 units. (I deliberately do not use the highly-recognized units of “decibels”, rather referring to an imaginary linear unit, since decibels use a logarithmic scale, which would make the example less easily appreciated). “Static” in this example can be seen as analogous to residual baseline confounding, and the sound volume without the static as analogous to the unconfounded treatment effect estimate.

### 1.2. A more complex, but more comprehensive, metaphor based on the recording of sound

The ACCE Method, as pointed out in the text, has a basic simplicity that can be represented in four steps or as few as two overarching components. As also pointed out in the text, however, these components (especially the first component) have a number of elements that still may be difficult for some readers to follow. To create a metaphor to more fully illustrate the important aspects of the proposed method, I provide a somewhat more complex example below. Hopefully, the payoff for this added complexity will be an intuitively understood, relatively nonmathematical picture of all of the important elements of the method.

Rather than basing an example on the *amplification* of sound, let us instead base it on the *recording* of sound. Assume now we are in the pre-digital recording era, when much recording of music occurs on cassette tapes. A taping companying has introduced a new tape recorder with a “noise reduction” feature. This noise reduction feature deliberately dampens down, or reduces, the white noise “hiss” that results from the operation of the internal machinery of the tape player. Prior taping equipment would introduce 11 units of this hiss into every 100 units of total sound they recorded. The new tape system only introduce 1 unit of this hiss for every 100 units of total sound recorded. The company’s engineers are very excited, and they make simple prediction. When we play an instrument at the exact same “volume” into the new equipment, we will get a recording with 90 units of sound, rather than 100 units. Not only will this lower volume be acceptable, but actually it will be highly desirable, because the recording will sound so much clearer and better. In fact, they expect people will rush to buy the new system.

Unfortunately, when the engineers test the first prototype of this new taping machine, they are thoroughly discouraged to notice that the instrument now registers as producing 105 units of sound, rather than 100 units, and the “hiss” is more audible than it had ever been with the old recording equipment. They are initially perplexed how this could happen when they were certain that the new system would reduce hiss from the working of the internal machinery by 10-fold. However, when they investigate their new system, they discover a new source of hiss from some degree of nonspecific deposition of the recording medium (e.g., iron filings) onto the tape surface due to electromagnetic fields generated during the taping process. This electromagnetic field causes some of the recording material to arrange itself incorrectly, creating this new source of “electromagnetic hiss”.

Their investigations subsequently show that somehow their new design, by seeking to minimize the noise introduced by the mechanical working of the tape player, unintentionally doubled the amount of hiss resulting from this 2 ^nd^ source of hiss. This discovery is so new that no one is even sure how much of this source of hiss is present in tapes. But the engineers are confident from their experiments that however much of this hiss exists, the new taping machine will exactly double this amount of hiss compared to the old taping system. (Let’s assume that in order to get the new machine to run the tape through especially quietly, the engineers had designed it with 4 recording heads, instead of 2, and it is known that each head will unavoidably introduce an undefined but highly consistent amount of electromagnetic hiss to the tape). The engineers realize that if they know the total volume of sound recorded by the 2 devices, they are now in a position to quantify the amount of the newly-discovered hiss originally present based solely on the information they have on hand. Here is how they would proceed.

The overall sound with the new recording system was unexpectedly amplified from 100 units to 105 units. They had expected the sound to go down to 90 units, because in their overly simple initial model they expected the only source of hiss was the 11 units of mechanical hiss introduced by the old system. Thus, they expected the original sound they were hearing was made up of 89 units of genuine instrument sound and 11 units of hiss. They had totally overlooked the second source of hiss -- because they had not known about it – and therefore had not factored it into their equations. They therefore expected the new system, with its only 1 unit of mechanical hiss, would have transmitted 89 units of genuine instrument sound and 1 unit of hiss, for a total of 90 units of sound. Armed with the knowledge that the new system, because it used 4 recording heads instead of 2, would be expected to double the 2 ^nd^ source of hiss (electromagnetic hiss), the engineers realized that the fact that the volume of the sound *increased* in the new system, rather than decreased, meant that quite a substantial amount of this electromagnetic hiss had to be present. A doubling of this electromagnetic hiss was more than enough to overcome the 10 unit reduction in the sound from the reduced mechanical hiss that they had expected. They determined that since they observed a 5 unit increase in volume rather than the expected 10 unit decrease, this meant that the doubling the electromagnetic hiss must have added 15 units of hiss to the sound: the 5 units increase in total sound they observed added to the 10 units of sound from mechanical hiss that their system had removed. (This equals a total of 15 units of sound). This calculation in turn would imply that there were 15 units of electromagnetic hiss in the original taping system that they had never noticed before (and certainly had never been able to quantify). For a doubling of this new source of hiss to increase the volume 15 units, it must have been originally present at an amount of 15 units.

In this example the genuine instrument sound the engineers are seeking to optimize represents the unconfounded treatment effect estimate. The mechanical hiss represents the Introduced Variable and its known effect on confounding (i.e., known “noise”). Its presence was not controlled in Recording System 1 (hence the contribution of 11 units of mechanical hiss sound), but its presence was highly controlled (and reduced) in Recording System 2 (only contributing 1 unit of sound). In a sense the engineer’s development of a noise reduction system largely removing the impact of mechanical hiss, can be seen as analogous to reducing the imbalance in the Introduced Variable by inserting it into the Model 2 regression. The ability of the engineers to predict the magnitude of decrease that occurs is analogous deriving a regression coefficient for the Introduced Variable(s) and predicting the change in the Model 1 treatment effect estimate through use of the Bross equation.

The electromagnetic hiss represents unmeasured confounders (i.e., unrecognized, unmeasured “noise”). The two systems represent Model 1 (the old system) and Model 2 (the new system). The new systems’ four recording heads relative to the two recording heads of the old system, and the predictable change in electromagnetic hiss that this change brings, represents possessing knowledge of the proportional amount of unmeasured confounding amplification expected to occur between the two models. Thus, being able to count that four heads are present instead of two is an analogue to the function of determining 1 - R ^2^ for the two exposure prediction models (or by using an “internal marker”).

What this example shows nicely is that if one wants to derive the true, genuine instrument volume of 74 units, one needs to take into account the amplification (i.e., doubling) of electromagnetic hiss in Recording System 2, but also to subtract the change in mechanical hiss from *both* the observed *change in total sound* from Recording System 1 and Recording System 2, and from the *original total sound value* for Recording System 1. (The genuine instrument volume would be equal to 100 units of sound minus the 11 units of mechanical hiss minus the 15 units of electromagnetic hiss detected by back-extrapolation from the increased hiss detected when this bias was doubled. That is, 100 - 11 - 15 = 74 units of sound).

This example helps illustrate key points. First, knowledge of the Introduced Variable effect (amount of mechanical hiss) is crucial, since without it, a very erroneous estimate of the quantitative effect of 2-fold amplification would result. If the reduction in mechanical hiss occurring in System 2 was somehow completely unaccounted for, then one would assume that a doubling of electromagnetic hiss that occurred led to merely a 5 unit increase in sound, and the value of the genuine instrument sound was 95 units (100 - 5) instead of 74 units. So correcting for the effect of the new, changed system (Recording System 2) on mechanical hiss is extremely important to obtaining an accurate estimate of electromagnetic hiss, and, by extension, and accurate estimate of the genuine instrument sound. Second, it is crucial to have a good estimate of the proportional amount of amplification of electromagnetic hiss. For example, if the two new heads were made of a different material than the other two, and this material had unknown effects on electromagnetic hiss, then the calculations given here could not be carried out.

There is a slight “difference in intent” between this metaphor and the actions being taken in the ACCE Method. The engineers only knew about one source of noise (the mechanical hiss), and sought to reduce this through their noise reduction system (our analogue for the Introduced Variable). Thus, they accidently, rather than intentionally, amplified the second source of noise, of which they were previously unaware. In biomedical or other intervention research, we know a second source of noise typically exists (unmeasured confounding), and we know the conditions that will amplify that second source of noise (through confounding amplification). Thus, in the ACCE Method we are using our two systems (models) to *intentionall*y produce amplification (specifically, to the degree possible, a *known* amount of amplification). In biomedical or other intervention research, the fact that we are likely reducing the impact of the first source of “noise” (mechanical hiss) is almost incidental, in that we are influencing the first source of noise only as the means to achieving a predictable change in the second set of noise. The effects of our Introduced Variable(s) on the first set of noise needs consideration solely so that an adjustment can be made for its effect, so as not to misestimate the change brought about through amplification of the second source of “noise” (and also so we do not misestimate of the total original noise present in our final calculations).

Despite this metaphor’s inherent greater complexity than the bacteria and sound amplification metaphors described earlier, this sound recording metaphor has at least two notable advantages. First, it fairly completely (and hopefully intuitively) represents each of the most important steps and concepts in the full ACCE Method. Second, if desired, it can be easily expanded to illustrate some current sources of uncertainty in the method (as illustrated below).

### 1.3. Expanding the recording metaphor to represent uncertainties in the operation of the method

To take the metaphor further, it is possible to use it to also illustrate some of the key sources of potential uncertainty in the ACCE Method estimate mentioned in the text (and elaborated on further in the Appendices that follow). If the increase in sound is unable to be accurately recorded beyond a certain volume, this might be seen as representing nonlinearities in the 1 - R ^2^ prediction of confounding amplification. If the inaccuracy at higher values further increased the volume of sound greater than expected, this could be analogous to what is observed in the Brooks and Ohsfeldt simulations above R ^2^ = 0.56 ^5^. If the inaccuracy resulted in lesser increases in the volume of sound than expected, this could represent real-world “constraints” to amplification, if they exist. If the engineers were able to identify some component of the sound (say, a certain wavelength of sound) whose change could be estimated accurately even in the presence of these nonlinearities (perhaps since its overall volume, representing only a small fraction of the total sound, was low) this might be seen as analogous to the “internal marker” strategy of estimating confounding amplification.

Also, consider if the old recording system and the new recording system also had other components which could contribute “noise”. For instance, what if the new recording system used jacks (plug-in connectors) on the end of its wires that were twice as large in diameter as the jacks used in the old system, and the larger jacks also reduced noise to an unrecognized extent? This would represent a source of altered noise separate from mechanical hiss or electromagnetic hiss. This can be seen as analogous to changes between the models that are not due to either confounding amplification or the effect of the Introduced Variable(s) which lead to treatment effect estimate differences. Obviously, such a “third source” of differences in sound volume would make accurate determination of the original sound present more complex. The easiest solution would probably be simply to design the two recording systems to use the same wires and jacks, so that this source of change in the noise recorded is unchanging. This is the basis of our recommendation to attempt to minimize other differences between the models, and/or to at least check to determine the extensiveness of these differences.

### 1.4. Summary

The sound recording metaphor shows that there may be reasons to suspect that confounding amplification in nonrandomized studies may bear useful similarities to the amplification of bias in other systems. Most importantly, our ability to quantify the original “genuine instrument sound” through the steps taken in this metaphor supports the possibility that the same basic approach (as laid out in the ACCE Method) should enable us to obtain an estimate of a genuine treatment effect.

The degree to which nonrandomized datasets actually permit straightforward quantification of unconfounded treatment effects through a procedure very similar to what was done in this very simple metaphor is not yet clear. Can we accurately estimate the proportional amplification we generate between two propensity score models as closely as knowing that four recording heads will double noise compared to two? Can we create our two models to minimize the chance that we distort our estimate of the unconfounded treatment effect by involving other changes that affect treatment effect estimates (similar to ensuring that the jacks at the ends of the wires were not changed between the Recording Systems)? The precise extent to which these or other aims can be achieved in the ACCE Method is uncertain, but research is clearly needed given the potential of the method to improve inferences from nonrandomized studies.

## Appendix 2: Other elements of the analysis that may produce changes in the treatment effect estimate between Model 1 and Model 2

This method ultimately views the change in the treatment effect estimate between Model 1 and Model 2 (minus adjustment for the contribution of the Introduced Variable(s) and their correlates) as arising from confounding amplification. As a result, an obvious and crucial need exists to keep all other differences between Model 1 and Model 2 to a minimum, to the extent feasible. Changes to the Model 2 treatment effect estimate, compared to Model 1, may potentially occur in several areas in addition to confounding amplification. As discussed below, these areas of potential differences between the two models include changes in the control of the confounding from “included” covariates (i.e., changes in the balance of covariates that are included in both propensity scores), the comparability of the patient sample, and the comparability of specific aspects of the intervention received by patients.

### 2.1 Differences in the balance observed for included covariates

Although these changes may be expected to be minor, at least in some settings, they deserve thorough consideration as part of an effort to anticipate sources of potential imprecision in the estimates resulting from the ACCE Method. Some degree of change between Model 1 and Model 2 is expected in the balance of each of the propensity score covariates present in common between Model 1 and Model 2 (the “included covariates”). (NOTE: The “Introduced Variable” is also an “included” covariate in a limited sense, in that it is included in one of the two propensity scores. For clarity in terminology and because the Introduced Variable represent a genuinely special circumstance [please see Appendix 3.2b], the term “included variable” or “included covariate” is reserved for the variables included in *both* models. The term “Introduced Variable[s]” is used for the variable[s] *added* to Model 2. The term “nonincluded covariate” will refer to covariates not included in either propensity score. Nonincluded covariates may either be unmeasured covariates, which inherently cannot be included, or measured covariates not selected for inclusion into the propensity score).

These changes are produced as a byproduct of the need to include an additional variable or variables in Model 2 to generate confounding amplification. For instance, including an additional variable in the propensity score used for matching would be expected to weaken at least slightly the tightness of the match on the other covariates. In some cases, the differences in covariate balance between models could be quite minimal. However, this balance needs to be explicitly compared between Model 1 and Model 2. This comparison is important since in other cases it may prove difficult to attain a degree of balance in the included covariates that is highly equivalent between Model 1 and Model 2 if sufficient confounding amplification is to be achieved. The impact of confounding amplification is to tend to create a greater number of individuals at the extremes of the propensity score distribution who are less comparable (and thereby, less similar in balance in the covariates included in the model) ^5^.

One approach worth consideration would be to examine whether it is feasible to adjust the stringency of the stratification or matching in Model 2 so that the balance in the included covariates in Model 1 and Model 2 are more equivalent. An alternative, and particularly rigorous, approach would be to use the Bross equation ^8^ to attempt to estimate the change in confounding attributable to the observed changes in the balance of each included covariates (even though this may represent a large number of covariates). (Going to the effort of applying the Bross equation to each of the included propensity score covariates potentially has an additional advantage, as discussed in Appendix 3.2a, in permitting the contribution to confounding from the residual imbalances in included covariates that are present in Model 1 to be estimated and removed from the Model 1 treatment effect estimate at the final stages of the process (please see Appendix Figure 1c).

### 2.2. Differences in patient sample

The overall patient cohort for the study from which the samples for Models 1 and 2 are derived will obviously not change. Some degree of change, minimal or otherwise, can be anticipated to occur, however, in the samples of individuals selected from that overall cohort by each model (Model 1 and Model 2). These differences can arise from differences in the patients that fall under the “Common Support Area” of the propensity scores, and, if matching is employed, differences in the percent of patients matched. The “Common Support Area” refers to the range of propensity score values which include members of both treatment groups; it is often recommended that individuals outside the Commons Support Area be “trimmed” (i.e., removed) from the analysis ^18^. These differences in patient sample, however, will only influence the method’s estimates to the extent that they are extensive enough to produce substantively different compositions of patients between the Models *and* effect modification exists (whereby the treatments studied have different effects in different patients). In addition, possible strategies exist to minimize some of these potential differences, as discussed below.

***2.2a. Differences in patient sample from differences in Common Support Area/propensity score trimming***. Because confounding amplification tends to make at least patients on the extremes of the propensity score distribution less comparable ^5^, it might prove difficult in practice to maintain a highly similar Common Support Area between Model 1 and Model 2. Fortunately, the number and identity of individuals differing between Model 1 and Model 2 is measurable. In addition, different approaches might be compared (such as examining only the subset of patients that fall under both model’s Common Support Areas). These comparisons might establish whether the results are sensitive to small differences in the Model 1 and Model 2 patient samples arising from different Common Support Areas.

***2.2b. Differences in patient sample from differences in percent matching***

Regarding matching strategies, it may prove difficult for a similar proportion of matching to be preserved between the two models. (The Brooks and Ohsfeldt simulation ^5^ showed that as unexplained variance of exposure decreased and amplification increased, the number of patients matched for a given caliper decreased). The alternative approach, propensity score stratification, may be determined to be the preferable choice for routine use in the ACCE Method, since by design stratification retains all individuals from the trimmed sample. (The ACCE Method emphasizes stratification and matching rather than weighting, because in the Brooks and Ohsfeldt simulation ^5^ propensity score weighting produced confounding amplification that was less predictable, at least by R ^2^).

As mentioned at the start of Section 2.2, these differences would only be expected to have relevance to the degree that effect modification existed (i.e., to the degree that the differences in patient sample would be expected to result in a change in the genuine treatment effect estimate between the two models). Because of the apparent dependence on effect modification for differences in Common Support Area or percent matching to impact the method’s performance, a separate approach to take to this issue would be to test for whether effect modification is detectable related to measured covariates. (However, this would obviously not address possible effect modification related to unmeasured covariates, although a consistent lack of observed effect modification might suggest a lower likelihood of effect modification than could be surmised before these observations). Clearly additional research (especially simulation research) is needed to assess the sensitivity of the method’s performance with and without the presence of treatment effect modification.

### 2.3. Differences in the specifics of the intervention received

While the general nature of the interventions received by the two treatment groups remains identical between Model 1 and Model 2, specific aspects of the intervention received can vary between the treatment groups in ways that are not immediately obvious. It is important to consider these possible differences because they may be another contributor to differences in the treatment effect estimates between Model 1 and Model 2 unrelated to confounding amplification.

***2.3a. Differences in dose***

Unless the intervention is a single, one-time-only dosed treatment, such as a vaccine, either dose or other “quasi-dose” aspects of how the intervention is administered may vary at least slightly between the individuals receiving the intervention in Model 1 and those receiving the intervention in Model 2. Even for nonmedication-based interventions, such as a psychotherapy or educational intervention, the timing of visits or the number of visits may vary slightly among the individuals included in the intervention arm in Model 1 versus Model 2. Therefore, when implementing the ACCE Method, it is important to examine whether the overall mean dosage, number, or timing of treatments is similar between Model 1 and Model 2, and potentially within strata for stratified analyses. If sufficient sample size exists in particularly large patient samples, an additional approach might be to restrict the analysis to patients only receiving one particular dosage of the treatment.

***2.3b. Differences in discontinuation rates***

Treatment effect estimates are likely to be sensitive to discontinuation rates, whether an intent-to-treat or an as-initially-treated analysis (i.e., with follow-up censored upon termination or alteration of the initial treatment) is conducted. Because of this, investigators should examine the rates of discontinuation observed in Model 1 and Model 2 to determine their similarity. Ideally, a determination that the *reasons* for discontinuation within the patient sample for Model 1 compared to Model 2 were also similar would be the ideal, but such information is often not available ^19^.

In many cases the difference in discontinuation rates for each treatment between the two models may be quite small, and the practical impact of this difference unclear. Differences in discontinuation rates appear to be at least slightly more significant in this approach, however, than in a propensity score or regression analysis involving a single model, *if* effect modification is present. In this case, differences in discontinuation rates between the two models could produce some degree of difference in the underlying treatment effect estimate between the models, even if no selection directly relevant to outcome was occurring.

Addressing specific differences (e.g., dose) in the intervention received by patients between the models, if they exist, may be at least slightly more challenging than minimizing differences involving confounding control from included covariates. One modest strategy might involve simply evaluating the differences in intervention specifics when different strategies are explored to minimize differences in Common Support Area or in the control of confounding from included covariates. Then the strategy that also minimizes differences in the intervention could be examined as the main analysis or as a sensitivity analysis.

As indicated in the main manuscript, this manuscript generally does not consider confounding arising after treatment initiation from differences in patient characteristics of patients who remain receiving in the two treatment groups. However, three points should be made. First, the “unconfounded treatment effect estimate” that is intended to be provided by the ACCE Method refers to an estimate unconfounded from baseline differences, but not necessarily completely unconfounded (i.e., it could still be confounded by differences arising after treatment initiation). Second, confounding after treatment initiation can exist, but if it is *similar* between the two Models, then it need not pose a barrier to obtaining a treatment effect estimate largely unconfounded from baseline factors. While the method is particularly dependent on the difference between Model 1 and Model 2 treatment effect estimates being attributable as much as possible to confounding amplification, in this case, confounding post-initiation would not be expected to be a major source of differences in the treatment effect estimates between the two models. In contrast, if substantially different amounts of confounding post-initiation exist in Model 1 and Model 2, this circumstance both produces a source of confounding not addressed by the ACCE Method and interferes with the ACCE Method’s ability to generate an accurate estimate of baseline confounding. At a minimum, rates of discontinuation should be checked to ensure they are similar between Model 1 and Model 2. Others have made the point, however, that similar discontinuation rates between treatment groups that are being compared can still conceal confounding after treatment initiation if the reasons for discontinuation differ ^19^. It remains to be seen, however, how often similar discontinuation rates between Model 1 and Model 2, which are likely to share a considerable number of individuals in common, in fact conceals differing reasons for discontinuation between the two models. (That is, the concerns raised in general about the possibility of different reasons for discontinuation existing despite similar discontinuation rates between treatment groups may not be as pertinent to the specific circumstance here of similar discontinuation rates between Model 1 and Model 2, since these models may share a substantial number of individuals in common). As mentioned above, the presence of similar discontinuation rates should be expressly confirmed. Further research is clearly needed.

Third, it is conceivable that the same approach used here – deliberately introducing confounding amplification to estimate the original confounding present – could be used, at least in theory, to also sequentially estimate residual confounding attributable to differential discontinuation during treatment. This is likely to be a substantially more difficult and complex endeavor than the use of the ACCE Method to estimate residual baseline confounding. For instance, the most commonly used approach for addressing measured confounding from differential discontinuation, generating a “pseudopopulation” by weighting, has been shown in simulation to produce confounding amplification that is considerably less predictable than matching or stratification ^5^. Re-matching patients after initiation could be considered, but the results would be expected to become applicable to only a smaller and smaller subset of patients. One relatively simple approach might be to conduct rematching at only a single additional time point: study completion. One challenge to using the ACCE Method to estimate confounding after treatment initiation may be that presumably the Introduced Variable(s) used to amplify confounding after treatment initiation will need to explain *differential* exposure to continued treatment (i.e., factors that lead to the discontinuation of one treatment but not the other). It may prove difficult to find variables that explain a substantial amount of continued exposure to one treatment but not the other (e.g., specific adverse events particular to one treatment might only affect a small percentage of individuals discontinuing treatment). This problem may be more easily addressable, however, if a set of variables can be introduced simultaneously ( Appendix 6).

Thus, while conceivably it could be ultimately determined that an assumption may need to be made for this method of “similar reasons for discontinuation between models”, it is too premature to make that conclusion. The possibility at least exists that this method, designed initially to address baseline confounding, may also allow residual/unmeasured confounding post-initiation to be addressed in those instances in which a suitable Introduced Variable or Variables for amplifying confounding post-initiation can be identified. Clearly, much additional work is needed in order to assess the feasibility of applying this approach to address confounding occurring after treatment initiation.

### 2.4. Summary

The need to evaluate, and potentially address, the diverse factors that may contribute to a change in treatment effect estimates between Model 1 to Model 2 initially may seem daunting. However, it may be ultimately determined that in practice little difference between Models in these aspects is typically observed. In theory, there may even be circumstances in which sizeable differences in some of these aspects do not prevent the method from providing accurate estimates (e.g., a difference in timing of an intervention whose effects have been shown to not be very sensitive to the timing of its administration). In most cases, however, substantive differences of the types described between the models are a concern. If these differences cannot be minimized by the strategies suggested, or approaches to quantifying the likely effects of these differences cannot be identified, then caution in interpretation is clearly warranted. Validation studies using simulated or real-world datasets would provide useful information concerning both the frequency with which these differences occur and their impact on the ACCE Method’s estimates.

## Appendix 3: Important considerations involved in the estimation of proportional confounding amplification

### Appendix 3.1. Approaches to estimating proportional confounding amplification

In the lower ranges of exposure prediction (at least as measured by explained variance in terms of R ^2^), a simulation has shown that a predictable relationship exists between amount of remaining unexplained variance in the prediction of exposure and confounding amplification ^5^. Differences in prevalence between treatment arms in covariates that are not included in the propensity score increase linearly with increases in R ^2^. This increase in the imbalance of uncontrolled (i.e., nonincluded) factors is the phenomenon that underlies the amplification of residual confounding. However, in the upper portion of the range of R ^2^ the relationship becomes increasingly nonlinear, with changes in R ^2^ underestimating the increased imbalance in nonincluded covariates ^5^. If this nonlinearity in the upper range of R ^2^ is replicated, but is reduced or not apparent for other metrics of prediction of exposure, then these metrics should be preferred. If this nonlinearity in the upper ranges of exposure prediction continues to hold for other metrics, then three strategies suggest themselves. The first approach, the “low amplification strategy”, would be to deliberately limit Models 1 and 2 so that the prediction of exposure these models achieve are in the lower end of the possible range, where the relationship is most linear. In some cases, propensity score models may already fall into this range. In other cases, this approach may involve reducing the variables included in the propensity scores. Such reduction might entail including only variables with a significant *a priori* expectation, based on evidence, of being confounders ^20^. An additionally restrictive strategy would including those variables estimated (by using the Bross equation ^8^) to be the most substantial confounders, or suspected *a priori* of being particularly certain or strong confounders (e.g., age, Charlson Comorbidity index, etc.). As discussed in Appendix 4, a particular high priority likely needs to be given to including variables for confounders that are correlated with the Introduced Variable(s), especially if they are strong confounders. Reductions in the number of included covariates could increase residual confounding relative to some other models that could be constructed, however, thus increasing reliance on the accuracy of the ACCE Method to address that increased confounding.

However, at least two other strategies suggest themselves that may prove feasible. One alternative would be to develop a formula that captures any nonlinearity in the chosen metric of exposure prediction. This could permit the amount of expected amplification to be relatively accurately predicted over larger portions of the range. The second alternative strategy would be to develop an “internal marker” covariate that would reflect how much increased imbalance in the nonincluded confounders is occurring. The internal marker would be a measured covariate deliberately left out of the propensity scores. The increase in its imbalance in Model 2 could be measured and serve as an indicator of confounding amplification.

Intuitively, an internal marker strategy has some attractive qualities, since it might sidestep any uncertainties about the relationship between confounding amplification and metrics of prediction of exposure. Furthermore, use of internal markers might prove the easiest way currently to apply this approach when logistic models are used to estimate exposure (as is commonly done for propensity scores).

There is already some evidence to support the “internal marker” approach. In the Brooks and Ohsfeldt simulation study ^5^ it was shown that covariates not included in the propensity score (and that are uncorrelated with the included covariates) all amplify to a remarkably similar extent, at least in that simulation. Thus, in principle, it appears feasible to use an “internal marker or markers” (produced by withholding measured covariates from the Model 1 and Model 2 propensity scores) to track and estimate the general amount of confounding amplification. A key practical consideration in real-world datasets, however, is the need for these internal markers to have a minimal correlation with any of the covariates included in the propensity scores. Any such correlations might “constrain” the ability of the internal marker to reflect the degree of confounding amplification that is influencing the nonincluded confounders. (These correlations, at least if positive correlations, would largely not be expected to interfere with confounding amplification for nonincluded covariates that are not being used as internal markers, for reasons discussed in Appendix 3.2). Since, in the strictest sense, some degree of correlation is virtually unavoidable, then the internal marker strategy may intrinsically underestimate true confounding amplification, although in some cases only minimally.

Fortunately, this correlation is readily measurable. Therefore, it should be possible to deliberately select the nonincluded measured covariate with the least correlation with both the included covariates and the Introduced Variable(s) to serve as an internal marker. Alternatively, simulation research may suggest quantitative approaches to correct for this correlation. If the internal marker strategy is used, this may constitute another practical reason to limit the covariates included in Model 1. In addition, should all candidate internal markers have some significant degree of correlation with covariates included in the propensity score, then any internal marker would be expected to underestimate the proportional increase in confounding amplification. Given the mathematics of the method, dividing a particular change in treatment effect estimates by an underestimate of proportional confounding amplification will overestimate confounding and lead to a conservative estimate of treatment effect.

In summary, multiple aspects of confounding amplification estimation are worthy of investigation. These include the presence and predictability of nonlinear relationships between the prediction of exposure metric and confounding amplification, and the potential strategies to address these nonlinearities.

### Appendix 3.2. An initial exploration of the impact of correlation on confounding amplification

Once the proportional confounding amplification has been estimated in general (through R ^2^ or other prediction of exposure metrics, or an internal marker), additional aspects of confounding amplification deserve consideration. These aspects center on the question of whether the predicted confounding amplification will in fact occur for all confounders.

The data that exists to date from the Brooks and Ohsfeldt simulation ^5^ indicates that confounding amplification is uniform between simulated covariates that were not included in the propensity score. However, this similarity may be a byproduct of their simulation. In theory, aspects of real-world data might create heterogeneities in amplification between covariates. The effect upon confounding amplification of correlations between covariates, expected to be a common feature of real-world data, is considered below. This conceptual exploration of the impact of correlations, which also draws upon the Brooks and Ohsfeldt simulation ^5^, tentatively concludes that many correlations do not appear to substantially interfere with the ACCE Method. Correlations can be categorized into five types, based on whether the correlated covariates are included or not included in the propensity score model: correlations between two nonincluded covariates, between a nonincluded and an included covariate, between nonincluded covariates and the Introduced Variable(s), between included covariates and the Introduced Variable(s), and between two included covariates. For the latter two categories, substantial amplification involving either of the correlated variables is not expected, at least in the same sense as the term applies to nonincluded covariates. (However, as pointed out in Appendix 2.1, the balance in included covariates can change at least somewhat between the two models and needs to be evaluated). Positive correlations between two nonincluded covariates also appear to often be nonproblematic. Both correlated variables would constitute part of the residual confounding being amplified, and thus be expected to be amplified to a similar extent, based on the Brooks and Ohsfeldt simulation ^5^.

A problem may exist, however, if some of the nonincluded variables are negatively correlated with some of the other nonincluded variables, since this might pose “constraints” to each amplifying to the extent predicted ( Appendix 3.3). Also potentially problematic, are correlations between nonincluded covariates and included covariates, and between nonincluded covariates and the Introduced Variable, although in the case of the former the impact is typically expected to be minor, and in the case of the latter, potential remedies may exist although these need to be evaluated. To explore the distinct issues raised by each of these classes of methods, we deal with each of these types of correlations as “special cases” below so as to explore the distinct issues raised by each.

***Appendix 3.2a. The special case of correlations between included and nonincluded covariates***

Correlations between included covariates and nonincluded covariates, however, could initially seem to pose the possibility of creating constraints to amplification for certain covariates. The measured covariates are included in the propensity score, cannot amplify substantially, and thereby might seem to constrain amplification, to a degree, of the correlated nonincluded variable. In fact, it is true that in this case the correlated nonincluded variable would be expected to have an *overall* change in imbalance in Model 2 that is less than the estimated confounding amplification. However, the implications of the Brooks and Ohsfeldt simulation ^5^ suggests that the change in a correlated variable can be alternatively modeled as a fraction that is largely unchanging (to the degree that it is correlated with included covariates that do not appreciably change), and a fraction (alternatively, a “residual”) that amplifies as much as any uncorrelated nonincluded covariate (Reference 5, Appendices). (These elements will be termed the “included fraction” and “nonincluded fraction” of the nonincluded covariate, respectively).

It is important to recognize that part of the goal for the nested models in the ACCE Method is to minimize change in the balance of included covariates, to the extent feasible ( Appendix 2.1). If the nested models do successfully exhibit little change in the balance of included covariates, then the Brooks and Ohsfeldt simulation ^5^ would suggest that the amount of imbalance observed in the “included fraction” of correlated unmeasured or nonincluded variables also would not change substantively between models. (The term “unmeasured or nonincluded” confounders or covariates will refer throughout these appendices to either unmeasured factors, which inherently cannot be included, or measured covariates that could potentially be included but are not included in a particular propensity score model). This lack of change in the included fraction would leave the amplification of the nonincluded fraction (which Brooks and Ohsfeldt have observed amplifies as completely as for the uncorrelated, nonincluded covariates) ^5^ as the only contribution to the *change* in treatment effect estimate attributable to this covariate. Thus, in principle, the method would still provide accurate final effect estimates of the confounding attributable to the amplifiable fraction of Model 1 confounding. However, a separate issue is raised by these included-nonincluded covariate correlations. A problematic “reservoir” of residual confounding would be built up that is neither part of the amplifiable fraction nor part of contribution to Model 1 confounding from the Introduced Variable(s) and its correlates. This “reservoir” is problematic, in theory, because even if an additional step to the method involving applying the Bross equation to each included covariate is added on, as described in the next paragraph, the bias from these correlates might prove to be only partially addressable.

As mentioned immediately above, consideration of the impact of included-nonincluded covariate correlations brings to the fore the fact that, even in the absence of any included covariate nonincluded covariate correlations, a small fraction of confounding would typically be expected to exist after application of the method attributable to the residual imbalance of the included covariates (since perfect balance in all included covariates is implausible). This residual confounding could be addressed in at least 3 ways. First, such confounding may be able to be minimized by achieving as close a balance as feasible between treatment groups for any variable included in the propensity score models. (This is yet another reason, in addition to those discussed in Appendices 3.1, to possibly favor smaller propensity score models, although again, this “adds pressure” on the method to deliver an accurate estimate of the now-larger residual confounding). Second, regression coefficients for each of the covariates in the model could be derived from multivariate models (including all of the other covariates) for each of the covariates in the model. The Bross equation could then be used to estimate the contribution of the remaining imbalance in each of these variables to residual confounding in Model 1 (in addition to accounting for any small change in balance in these covariates observed in Model 2 compared to Model 1, as discussed in Appendix 2.1). For investigators particularly interested in obtaining the most rigorous unconfounded treatment effect estimate available through this method, such an approach may be preferable to performing no adjustment, even if these efforts change the unconfounded treatment effect estimate only modestly or minimally (Please see Appendix Figure 1c). Third, all of the included propensity score covariates could also be inserted into the Step 1 treatment-outcome regression equation estimating the Model 1 and Model 2 treatment effects (i.e., in *addition* to including these covariates in the propensity score). This should largely address residual confounding arising from the remaining imbalance in these included covariates (again, this might pose some upper limit on the number of covariates that could be included in propensity score). However, it should be noted that the degree to which either the Bross equation approach or the insertion of the correlated included covariate into the treatment-outcome regression fully corrects for the impact of residual confounding from correlated nonincluded confounders is uncertain. Confounding amplification relating to the estimation of regression coefficients for the included covariates could also conceivably be a problem. Therefore, it is uncertain whether attempting to minimize covariate imbalances between the treatment groups or address those differences through one of the two regression-based approaches should be clearly preferred. On the other hand, it must be pointed out that we are considering the impact of a fraction of what already is a fraction of the confounding from the nonincluded covariate. That is, we are considering the residual “reservoir” of confounding from the included fraction of the nonincluded covariate that is not addressed through the Bross equation or insertion in the main regression. This residual is itself just a fraction of the “included fraction”, which already is a fraction of the overall confounding from the nonincluded covariate. It therefore may prove that, in a practical sense, this concern is typically not a major problem. It could be argued, however, that the sum of a larger number of these minor residuals might have some noticeable impact on the treatment effect estimate. And that the uncertainty around the impact of this element of confounding is concerning because, strictly speaking, its size cannot be easily estimated. This may constitute one aspect by which the method may provide an estimate of residual confounding that is at least slightly incomplete; that is, this method may underestimate residual total confounding to some degree in typical practice. Further research regarding this issue would be clearly beneficial.

***Appendix 3.2b. The special case of correlations between nonincluded or unmeasured covariates and the Introduced Variable(s)***

Correlations between nonincluded covariates and the Introduced Variable are a particularly distinct circumstance. In this case, the Introduced Variable is an included variable in only one Model (Model 2). As a consequence, its balance is being deliberately changed from Model 1 to Model 2 to produce the needed confounding amplification. The “included fraction” of any nonincluded covariate that relates to its *correlation with the Introduced Variable(s)* is therefore being more closely balanced in Model 2 than in Model 1. This is because in Model 1 the nonincluded covariate’s correlate, the yet-to-be-inserted Introduced Variable, is not controlled at all, except for any control related to correlations between the Introduced Variable and included covariates, which stay largely constant between Model 1 and Model 2. (To clarify, the set of variables in both models that are the included covariates stay constant, but the exact balance of these variables may vary slightly or somewhat between the models). However, it is partly for this circumstance that the Step 3 and 4 procedure was designed involving deriving regression coefficients and applying the Bross equation ^8^. The intent of Steps 3 and 4 is that the change in confounding of the Introduced Variable is estimated, *along with,* to some degree, the change in confounding resulting from the change in imbalance in the fraction of the correlated nonincluded variable(s). Whether the effect of correlation upon regression coefficients, however, is sufficiently similar to the effects of correlation on the balancing of covariates using a propensity score to permit a generally effective adjustment is uncertain.

This is an area of the ACCE Method in which further research would be particularly beneficial. The comparability of the quantitative effects of correlation on regression coefficients versus on covariate balance in propensity score analyses could be examined further through simulation, and perhaps theoretically through frameworks based on the general location model ^21^ or other methods. Such simulations would helpfully allow the strength of the correlation between correlated variables and the amount of confounding amplification existing between the two models to be varied. Real-world studies could contribute by investigating how frequently the ACCE Method provides what appears to be improved treatment effect estimates (i.e., closer to the result that is expected based on randomized trials) ^16, 17^ than typical propensity score or regression methods.

Finally, any imprecision in the ability of the adjustment in Steps 3 and 4 to adequately reflect the change between the models in confounding attributable to the nonincluded covariates correlated with the Introduced Variable would depend on the number and strength of such correlations. While this is impossible to quantify the correlations present for truly unmeasured covariates in real world data, it may turn out, based on the comparisons to randomized data discussed above, that in practice these correlations often do not appear to be numerous or strong enough to substantially affect the method’s estimates. Even if the regression coefficient/Bross equation-based adjustment was ultimately shown to only poorly capture the effects of correlation, it is possible this may not interfere markedly with the overall accuracy of the method if most of the residual confounding is not correlated with the Introduced Variable. As discussed in Appendix 4.1, it may be possible to test for this correlation, even though the correlation involves unmeasured confounders, by a particular re-application of the ACCE Method.

Even in the “worst case” scenario in which the adjustments in Step 3 and 4 of the method do not perform well *and* comparisons with randomized data suggested that this limitation typically impairs the method’s estimates substantially, three special circumstances exist in which the ACCE Method’s performance would not be generally expected to be adversely impacted by this limitation. These special circumstances would include using as Introduced Variables either 1) true instrumental variables (although it is uncertain whether any significant advantages would exist for the ACCE Method compared to conventional, 2-stage instrumental variable analysis); 2) near-instrumental variables, or 3) a related, but less restrictive, category of variable: variables with an independent association with outcome but little correlation with other confounders. As long as the Bross equation ^8^ adequately captured the effects upon confounding of increased control of this outcome-associated but uncorrelated-with-other-confounders type of Introduced Variable upon confounding, then imprecisions in how the regression-coefficient based adjustment in Steps 3 and 4 captured the effect of correlated covariates would be relatively immaterial (since little correlation would be present). The frequency of such variables, however, is unclear. In addition, as pointed out above, it is impossible to determine conclusively whether a variable is correlated with unmeasured confounders in real-world data. As we describe in Appendix 4.1, however, one approach may exist to attempt to test for a lack of correlation between the Introduced Variable and unmeasured or nonincluded confounders of the treatment effect estimate. Another, likely less powerful, approach towards applying this variant would be confirming its lack of significant association with *any* the measured covariates available (although a lack of correlation with measured covariates certainly could not be taken as conclusive evidence of a lack of correlation with unmeasured covariates).

In addition, even if the ACCE Method was ultimately determined to typically provide only substantially imprecise estimates of total residual confounding, several beneficial applications suggest themselves. The first is simply determining the *direction* of the remaining residual confounding in an association after efforts to control for confounding, which sometimes can differ from the direction of initial confounding. Second, even imprecise estimates from the method may be able to provide indication of whether residual confounding appears to be *a small, moderate, or large* contributor to the observed treatment effect estimate. Along similar lines, associations between treatments and multiple outcomes could be able to be investigated, with the method providing useful information concerning which associations between a particular treatment and a variety of outcomes appear to be the least confounded, even if a precise estimate of this confounding cannot be obtained. For these reasons (as pointed out in the text) this method may be a particular benefit to database surveillance research that seeks to identify promising associations for further detailed investigation. Lastly, by at least partially concentrating uncertainty concerning residual confounding to a particular focus upon the Introduced Variable(s) and its potential correlates, the method may permit, in some instances, a beneficial focusing of future investigations. This may be helpful, for instance, if additional information about the Introduced Variable’s anticipated correlates can be easily gathered (e.g., through chart review), thus moving this correlate, to some degree, from “unmeasured” to “measured.”

While many possibilities can be anticipated theoretically, a clearer answer concerning the significance of issues around potential correlations involving unmeasured or nonincluded covariates will likely await both simulation studies and empirical testing of the method on real-world data in which the presumed genuine treatment effect is known. For example, a fraction of the 234 “negative controls” ^17^ identified by The Observational Medical Outcomes Partnership for which there is particular confidence about the lack of association might provide a very useful “substrate” to test the method’s ability to remove residual unmeasured confounding and provide unconfounded treatment effect estimates.

### Appendix 3.3. The possibility of additional “constraints” upon confounding amplification

Another important issue to consider is the possibility that, in real-world datasets, inherent limits or “constraints” may exist to how much a covariate can conceivably amplify regardless of changes in exposure prediction. Two such possible constraints have been already mentioned in Appendix 3.2: the “constraints” possibly imposed by a negative correlation between two nonincluded confounders, and by nonincluded covariate-included covariate correlations.

Initially, the possibility of a different set of constraints might also seem plausible, based on limits to how much an imbalance in a particular covariate could inherently change. Such a concern might seem plausible, for instance, if the sample matched on the propensity score already included almost all individuals possessing this characteristic. However, further consideration suggests that such seeming “practical constraints” may not interfere greatly with the method in practice. While such a constraint presumably would limit how much the prevalence of that specific covariate in a treatment group could *increase*, it would not limit how much the prevalence of that covariate could *decrease* in the opposite treatment group. Since the imbalance between treatment groups relates to the relative difference in prevalence between the groups, such an imbalance could occur as easily through a loss of representation of the covariate in one group as from a gain in representation in the other. One obvious, although potentially modest worry, however, would be whether such a decrease in the prevalence of a covariate would come at the cost of creating a substantial difference in the Common Support Area or percent matching between the propensity score models. (If so, this would represent one of the potential differences in the patient sample between models discussed in Appendix 2.2).

Among the most substantial concerns regarding possible “constraints” is the issue that was noted in passing in Appendix 3.2: negative correlations between two nonincluded or unmeasured covariates. Covariates are not free to change in isolation. For instance, let us assume that, for a medication study, Model 2 confounding amplification is supposed to double the imbalance in two unmeasured confounders: recent adverse effects from surgery and health care access. Assume as well that those individuals at greatest risk for experiencing adverse events from surgery in the study period are, on average, also those individuals with superior health care access. Now assume that in the treatment group there is a greater prevalence of adverse events from surgery but, for unclear reasons, a lower degree of health care access than in the comparison group. For confounding to double for both covariates, a greater difference in the number of individuals with adverse events from surgery needs to be present (with more of these individuals in the treatment group), along with a greater difference in the number of individuals with health care access (with fewer of these individuals in the treatment group). Thus, it appears difficult, solely through adjusting the prevalence of individuals with adverse events and superior health care access to achieve a worsening of both of these imbalances. Conceivably, this means that the difference between the treatment group in the specific fraction of individuals with just adverse events, or just superior health care access, might be particularly amplified, but this remains purely speculation until simulation can address this issue.

It is also difficult to predict the routine plausibility of this scenario (in which many of the individuals in a treatment group who exhibit one confounding characteristic also possess a second confounding characteristic, yet a negative correlation between these variables exists overall). Once again, since the degree of imbalance in the truly “unmeasured” confounders can clearly be specified only in simulation (although mock “unmeasured confounders” can be created in real-world studies by deliberately not including some measured covariates), the first step to assessing the implications of these scenarios would almost certainly be through simulation research.

In the meantime, I have sought to capture these potential uncertainties in the manuscript through the use of the general term “the predictability of confounding amplification”. Uncertainties about the predictability of confounding amplification could fall into at least four general categories. One potential category would be if the change in prediction of exposure (or change in an internal marker) failed to correlate well with the overall confounding amplification observed (i.e., imprecision in the estimation of proportional confounding amplification). A second potential category would be if different unmeasured confounders simply inherently amplify to different extents for a given change in the prediction of exposure (i.e., there is an inherently wide variability in how much individual confounders amplify, even if they completely lack correlations). The third potential category relates to the possibility that unmeasured confounders may amplify essentially as predicted until a certain point of exposure prediction when “constraints” appear to manifest themselves and progressively impede amplification. The final category would relate to the impact of correlations on the method's estimates.

It should be pointed out that if real-world “constraints” to amplification exists, they would be generally expected to produce opposite effects with increasing prediction of exposure to what was observed in the Brooks and Ohsfeldt simulation ^5^. That is, real-world constraints in the amount that a confounder could amplify would become progressively evident at higher ranges of exposure prediction, when confounding amplification is expected to be greater. As a result, presumably result in less change in the Model 2 estimate would be observed than predicted by a 1 - R ^2^ amplification of confounding, rather than the greater than expected change observed by Brooks and Ohsfeldt above an R ^2^ of 0.56 ^5^. As an obvious corollary, if constraints to amplification exist then their effects are likely to be less evident at lower levels of amplification, for which less change in the balance of unmeasured covariates is expected in Appendix 3.1 and Appendix 3.2. Thus, this consideration may have similar implications to others previously mentioned in support of working in the lower ranges of exposure prediction. Nevertheless, the adoption of such a radical strategy would need to be supported by considerable research supporting this method’s quantitative accuracy, especially since it may prove more difficult to the lower range of exposure estimates to accurately discern differences between treatment estimates.

### Appendix 3.4. Other considerations, such as the impact of the form (exponential versus linear) of the treatment effect estimate

Other important considerations in the application of the ACCE Method can be envisioned. To provide one example, since the method involves the straightforward subtraction of one treatment effect estimate from another, it is possible that the noncollapsibility of odds ratios and hazard ratios will be problematic. That is, noncollapsibility may provide another source of difference between the Model 1 and Model 2 treatment effect estimates (i.e., a difference not related to confounding amplification). (As mentioned previously, a key requirement of this method is that the difference between the Model 1 and Model 2 treatment effect estimates represent, to the extent feasible, only the effects of amplified confounding). Therefore, it may be determined that this method works better for linear outcome models (such as those involving either continuous outcomes, or probabilities of the outcome rather than the presence or absence of the outcome itself), or log-linear (e.g., log-binomial, Poisson) outcome models (which provide risk ratios) than logistic outcome models. This manuscript already focuses upon using linear, rather than logistic, propensity score models to allow for estimation of the change in the prediction of exposure. This choice was made simply because the linear model is the model for which the work on the quantitative relationship between changes in prediction of exposure and confounding amplification has been the most developed ^5^. Obviously, there are well-known shortcomings of using probabilities for dichotomous healthcare endpoints such as treatment, mortality, or disease onset. Nevertheless, some other innovations in nonrandomized treatment research have been introduced in the form of their application to linear regression models ^22^. The best modelling approaches to pursue for both treatment exposure and outcome to optimize the performance of the ACCE Method are not yet clear.

## Appendix 5

This appendix consists of three figures. The first figure ( Appendix Figure 1a) annotates (i.e., labels) the components of the ACCE Method Summary Equation. The second figure ( Appendix Figure 1b) indicates to which terms the various uncertainties discussed in the main manuscript and these appendices pertain. The third figure ( Appendix Figure 1c) adds terms relating to the change in imbalance in the *included* covariates between Model 1 and Model 2, and the residual imbalance in *included* covariates in Model 1. These terms may be minor and thus adjustment for them through using the Bross equation not always needed. In theory, however, such adjustments should provide more optimal estimates.

## Appendix Figure 1b Legend: Key Uncertainties in the ACCE Method mapped to terms in the Summary Equation

1. Will the effects of random variation routinely cause sufficient misestimation of this difference to impair the method’s usefulness? To the extent this problem exists, it would be expected to decrease as the size of the database increased. In addition, the significance of the problem may also be diminished by serial estimations of the Treatment Effect Estimates, such as through bootstrapping. This would not reduce random variation, per se, but would minimize the chance a single particularly inaccurate estimate becomes the basis for all the conclusions about unmeasured confounding. As pointed out in the manuscript, some groups have reported results suggesting that very small differences in treatment effect estimates between similar models can be reliably detected ^9, 10^.
2. Given uncertainty #1, in general it would seem desirable to maximize the amount of difference between the two treatment estimates (i.e., maximizing confounding amplification between the two models) to maximize the chance of detecting a “signal” within the “noise” of random variation. However, this principle may be in opposition to Uncertainties #3, #5, and #10.
3. Are the two propensity score models as similar as feasible in other aspects? Specifically, is the balance for the measured covariates included in both models similar between both models? Is the Common Support Area and, if applicable, percent matched similar? Are aspects of the intervention, such as the average dose and frequency of different doses across the dose range for the treatment groups (and the rates of discontinuation of the intervention and the comparator) similar between the two models? Of note, the second concern regarding the Common Support Area, percent matched, etc., only becomes relevant to the extent that the treatment examined exhibits effect modification between the two groups (i.e., that those differences in the groups’ composition affect the underlying genuine treatment effect).
4. Given that a core operation in the method involves subtracting two treatment effect estimates, are linear or exponential models to be preferred? This question is relevant because of the possible impacts of issues such as noncollapsibility and linearity assumptions on the accuracy of the two treatment effect estimates as well as the subtraction of these two treatment effect estimates. It is important that, to the extent feasible, the difference in effect estimate only reflect differences in confounding amplification between the two models, not other differences (including differences attributable to misestimation).
5. Does the Introduced Variable – outcome regression coefficient, when inserted into the Bross equation, adequately reflect the changes that are also occurring in correlated unmeasured confounders? That is, these correlated unmeasured covariates would generally be expected to have an overall amplification less than predicted by (1 / (1 - R ^2^_M2_)) / (1 / (1 - R ^2^_M1_) due to their partial correlation with the Introduced Variable. The Brooks and Ohsfeldt (2013) simulation ^5^, however, suggests that this can be modeled as a certain fraction of the unmeasured confounder coming into approximately the same balance as the Introduced Variable (as predicted by the correlation coefficient) while the remaining fraction amplifies to the same extent as the rest of the unmeasured confounders. Would using an Introduced Variable-Outcome regression coefficient (that would be expected to be affected by the correlation between the Introduced Variable and the correlated unmeasured confounder) as the input for the Bross equation adequately estimate the change in confounding occurring when the Introduced Variable(s) is inserted into Model 2 (i.e., Conf _IntV _Δ(M2-M1)__)? In addition, we must rely on the subtracted term representing the Introduced Variable(s)’s contribution to Model 1 (i.e., Conf _IntV _M1__) to also capture the confounding contributed by the nonamplified fraction of the correlated unmeasured confounders.
6. The Introduced Variable – outcome regression coefficient potentially may suffer at least some degree of confounding amplification, although it is possible that in most cases this amplification is not as severe as that which occurs in the treatment-outcome relationship. In addition, it may be possible to identify cases in which significant unmeasured confounding is unlikely, although how frequently this occurs is uncertain. Finally, it may be possible to reduce this potential bias by repeating the estimation using yet another model and applying the ACCE Method to predict the magnitude of confounding amplification in the Introduced Variable-outcome regression coefficient.
7. Is the Bross equation straightforward approach of using a regression coefficient multiplied by the observed imbalance in a variable truly strictly accurate in accounting for that variable’s contribution to confounding?
8. Although there is some simulation and theoretical work supporting the 1 - R ^2^ confounding amplification relationship, more evidence supporting this relationship would be desirable. In addition, the simulation work, but not the theoretical work, demonstrates increasing nonlinearities in the 1 - R ^2^ value versus the actually observed confounding amplification, raising a question of how accurately the 1 - R ^2^ relationship predicts amplification above R ^2^ of 0.56. It should be noted, however, that if a predictable relationship between R ^2^ or other predictors of exposure and confounding amplification is not possible, use of an “internal marker” strategy might still allow the method to be implemented.
9. A key assumption of the method is that different unmeasured confounders amplify uniformly or reasonably uniformly, but other than a single simulation it has not been demonstrated that this is the case. Specifically, the question is whether unmeasured confounders that are uncorrelated with the included covariates or Introduced Variable(s) amplify uniformly (and potentially, as discussed in #5, the fractional component of correlated unmeasured confounders that can be represented as not correlated with the Introduced Variable or included covariates). One possibility, for instance, is that in real-world data “constraints” may exist pertaining to the actual amplification that can be achieved for different unmeasured confounders.
10. Are there advantages in keeping expected confounding amplification relatively low to minimize the possibility of exceeding the R ^2^ = 0.56 “threshold” (if this threshold in fact does exist), minimizing the likelihood of constraints, etc. If so, this would be expected to work at cross-purposes to the strategy to address point #2: maximizing the difference between the treatment effect estimates to facilitate the most accurate estimates of residual confounding.

## Appendix 6. Considerations involved in adding a set of Introduced Variables to induce substantial confounding amplification

One aspect of the ACCE Method that deserves further investigation concerns whether the insertion of a set of Introduced Variables, rather than a single Introduced Variable, could be used to generate confounding amplification. Use of a set of variables would remove the need to identify a single variable with a strong association with exposure, potentially greatly enhancing the applicability of the method. Indeed, datasets which lack a single variable with a strong association with exposure to serve as the Introduced Variable could presumably be “engineered” in most cases to have that function played by a set of variables. That is, a very large number of datasets would be expected to contain enough measured variables to, in total, predict exposure to some substantial amount when inserted as a set. These variables could simply be withheld from Model 1 and inserted in Model 2. In some extreme cases, this might require withholding virtually all of the measured covariates from Model 1, so that they could serve as the Introduced Variable set inserted in Model 2. Many of these variables might have associations with outcome, but that would be addressed through application of Steps 3 and 4 of the ACCE Method for each inserted variable.

Such an approach of deliberately withholding a set of Introduced Variables, however, incurs the potential drawback of placing increased emphasis on the ability of the ACCE Method to accurately quantify residual confounding, as discussed in Appendix 3.1, Appendix 3.2, and Appendix 3.3. However, the potential to derive some sort of estimate of unmeasured confounding in an extremely large number of studies might be a sufficient advantage to counterbalance or outweigh this concern.

In theory, there appears to be no mathematical reason mathematically why multiple Introduced Variables cannot be used. For the simplest example, consider two variables, each present at an 80%/20% prevalence in the two comparison arms (treatment and control), one that had an association with outcome of beta = +0.1, and the other an association of beta = -0.1. (Presumably each of these coefficients would be obtained from a regression including all the other propensity score covariates, including the other Introduced Variable). Upon insertion, if both covariates were also essentially equally balanced in Model 2 (e.g., both become balanced at a 52%/48% prevalence), then seemingly they would serve as a composite Introduced Variable with no overall association with outcome, but which would amplify confounding more than either variable alone.

In the more complex case in which the associations of the Introduced Variables did not cancel out, presumably Steps 3a–c (e.g., derivation of the regression coefficients and use of the Bross equation) would be performed for each of the Introduced Variables (as mentioned above). Then each of the changes in confounding attributed to the Introduced Variables would be subtracted from the overall change in the treatment effect estimate observed. Likewise, Step 4b1 would subtract the confounding attributable to each of the Introduced Variables from the Model 1 treatment effect estimate.

One concern, however, would be, as the number of Introduced Variables increases, the likelihood would increase that at least some Introduced Variables would be associated with unmeasured confounders. The test for whether that is the case proposed in Appendix 4, however, presumably still holds. The feasibility, advantages, and disadvantages of using a set of Introduced Variables still need to be tested out in simulation. Nevertheless, the use of a set of Introduced Variables would seemingly create, in theory, the potential to obtain a quantitative or at least a qualitative sense about residual confounding from a large number of intervention studies.

## Appendix 7. Practical guide to implementing the method based on current knowledge and final thoughts

### 7.1. Tentative suggestions for implementing the ACCE Method based on current knowledge

Much simulation and real-world research needs to be performed to evaluate the degree to which the ACCE Method provides valuable estimates of unconfounded treatment effects, or at least estimates closer to the likely unconfounded treatment effect than the conventional estimates obtained prior to application of the method. Such research may include potential “tuning” of the method to simulated or real-world datasets through determining the optimum choices for the method’s implementation, either in general or in specific circumstances (see below, and preceding Appendices). Research of this type must occur before strong recommendations can be advanced concerning preferred approaches to use in implementing this method. Such research would also clarify the weight that should be given from estimates from this method compared to other methods for addressing unmeasured confounding. Hopefully, the method has been communicated in the manuscript and these Appendices clearly enough that researchers can start to implement the method in a variety of datasets to determine, and determine how to optimize, its performance.

Nevertheless, some researchers may understandably have a current interest in preliminarily exploring what this method suggests about the likely presence and size of unmeasured confounding in their analyses. A few key considerations suggest themselves:

First, there appear to be three key qualities for the Introduced Variable or Variables:

1) **Strength of association with exposure** – all other things being equal, stronger is better.
2) **Likelihood of, or evidence for or against, correlation with other confounders (especially unmeasured confounders)** – all other things being equal, fewer or no apparent correlations is desirable.
3) **Association with outcome** – all other things being equal, a lack of an apparent association with outcome is preferred, although far from required.

The ideal Introduced Variable would presumably possess both attributes #1 and #2 (a strong association with exposure and little to no apparent correlation with other unmeasured confounders). The association with outcome (#3) is expected to be less crucial, especially if a lack of correlation with other unmeasured confounders is suspected. In this circumstance, the effect upon confounding of adding this variable into the propensity score, regardless of its association with outcome, will hopefully be adequately captured by the regression coefficient/Bross equation-based adjustments outlined in the manuscript. However, as discussed in Appendix 4.2, at this point the method’s estimates probably should be seen as at least slightly more uncertain whenever a substantial quantitative adjustment to the Model 1 treatment effect estimate has to be performed. In other words, the method may allow for the most confident conclusions to be drawn when the method suggests little residual confounding exists.

Among the first two attributes listed, it is difficult to offer definitive advice about which to prioritize until the method’s performance in the context of substantial random variation is determined. If random variation represents a major threat to the accuracy of the estimates resulting from the method, then high priority will likely need to be given to adding an Introduced Variable or set of Introduced Variables that is strongly associated with exposure. This presumably would produce the greatest expected change in the treatment effect estimate, likely increasing the chance of detecting a relatively accurate change in the setting of substantial random variation. Similarly, research is also needed to determine the extent to which the proposed adjustments to quantify the effect of inserting the Introduced Variable into the second model also successfully captures the change that results in other, correlated, unmeasured confounders. Until that point, it would appear that choosing an Introduced Variable with little or no apparent correlation with other unmeasured confounders, if one or more can be identified, would be advisable. How frequent such variables are routinely available is uncertain. It may prove possible, however, to screen for such variables through selection procedures based on detecting an apparent lack of change in the Introduced Variable(s)-outcome association when unmeasured confounding amplification is expected (this procedure is broadly outlined in Appendix 4).

The lack of association with outcome appears to be a less important consideration than the preceding two. However, if it is difficult to demonstrate with satisfaction that an Introduced Variable appears uncorrelated with unmeasured confounders, than there may be an advantage to choosing an Introduced Variable with a minimal association with outcome. At a minimum, such a strategy would appear to have a reasonable chance of minimizing confounding amplification of the Introduced Variable-outcome association, especially if there were no *a priori* reasons for believing the variable would have an association with outcome. A variable thought to have no association with outcome that has a regression coefficient close to the null likely has a better chance of having minimal correlation with unmeasured confounders than one with an observed association with outcome, although it is always possible that the apparent null association results from an Introduced Variable with a substantial association with outcome closely counterbalanced by correlated unmeasured or nonincluded confounders biasing in the opposite direction. Again, however, the test of the sensitivity of the Introduced Variable coefficient to the number of variables in the model proposed in Appendix 4.1 might permit the evaluation of that possibility (by determining if substantial correlations with unmeasured or nonincluded confounders are suggested.

Whether using Introduce Variables with a null or near-null association with outcome is any advantage over conventional instrumental variable regression, however, is uncertain. It is conceivable that using the ACCE Method with such variables may provide additional statistical power over a conventional instrumental variable regression, and/or may not as prone to the biases that weak instruments can engender (especially if the use of a set of Introduced Variables produces stronger changes in the prediction of exposure). These are untested possibilities, however, and await both systematic investigation of the method’s performance in general and development of methodology to generate confidence intervals via bootstrapping or other approaches.

In addition to the qualities of the Introduced Variable, at least one additional major strategic consideration will likely influence ACCE Method model-building decisions: the judgment of how extensive Model 1 should be (i.e., the number of covariates it contains). In general, there are obvious advantages of having the model be as extensive as possible (i.e., to adjust/balance for as much measured confounding as possible). Methods for minimizing measured confounding in such a fashion have been developed for decades, while the ACCE Method has just been proposed. A second clear-cut reason for attempting to control as much measurable confounding as possible is that such a strategy reduces the risks that the nonincluded covariates will represent “unmeasured confounding” that is correlated with the Introduced Variable(s). Since the method’s performance in the setting of such correlations is more uncertain, it makes sense to attempt to construct the models to avoid this condition as much as possible. Certainly, at a minimum, it would seem like any known confounders that clearly are correlated with the Introduced Variable should be included as propensity score covariates. A third reason to maximize the number of included covariates is that in general, the greater degree to which exposure is predicted, the larger the predicted amount of confounding amplification between Model 1 and Model 2. This larger proportional confounding amplification means that the quantitative estimate of confounding amplification, with its associated uncertainty, is multiplied by a lesser factor than if little difference in proportional confounding amplification is observed. (For an extreme example, if two models are compared, one with an R ^2^ for exposure of 0.1 and one with an R ^2^ of 0.2, this means the quantitative change in treatment effect estimates would be divided by (1 / (1 - 0.2) / 1 / (1 - 0.1)) - 1 = ((1 / 0.8) / (1 / 0.9)) - 1 = 1.125 - 1 = 0.125. Dividing the change in treatment effect estimates by 0.125 is the equivalent to multiplying this change by a factor of 8! In general, it seems more prudent to, if the opportunity exists, work in a higher range of exposure prediction where the treatment effect estimate difference is multiplied by a much lesser factor).

However, despite the attractiveness in general for making Model 1 as complete as possible, there are a couple considerations which may, in certain circumstances, support creating a Model 1 that is deliberately less extensive than might be possible. The first consideration is the simple reality that the Introduced Variable association with outcome needs to be determined via regression. The number of variables that can be inserted into a regression equation depends upon the number of outcomes, while the number of covariates that can be introduced into a propensity score is often far greater, since this quantity relates to the total number of individuals exposed. Thus, in settings with relatively few outcomes, the number of covariates that can be entered into a propensity score may easily exceed those that can be included in a regression without risking bias. Second, if it is determined that the apparent nonlinearity that starts to affect the confounding amplification relationship above an R ^2^ of 0.56 in simulation is genuine, and this nonlinearity cannot be modeled mathematically and/or reflected in the change in “internal markers,” then a strong reason might exist to keep the number of propensity score covariates in Model 1 smaller than otherwise might be possible. A need would exist to keep the Model 1 propensity score covariates at a number for which addition of the Introduced Variable or variables in Model 2 does not produce an R ^2^ greater than 0.56. However, it is not clear whether this observed nonlinearity relates to some specific characteristics of this simulation. Third, the possibility of “constraints” upon confounding amplification is another possible reason for limiting the number of covariates representing measured confounders in the Model 1 propensity score. To the extent that some level of “constraints” to confounding amplification occur within real-world data (which is currently unclear), the general expectation would be that these constraints would be less pronounced at lower R ^2^ values of the two models (since less overall amplification would likely be resulting) than higher R ^2^ values. Finally, it is worth considering the desire to keep Model 1 and Model 2 as comparable as possible except for the Introduced Variable. Differences between the models in balance of measured confounders, patient sample, or dose or duration of the intervention could produce different underlying “treatment effects” in the two models, although how large these differences would typically be is uncertain. In general one would expect that as the amplification becomes greater between the two models, the potential for a difference in these factors becomes greater. These differences can be measured, however, and it may prove that this consideration is relatively minor compared to, for example, the advantage of substantially amplifying confounding in the setting of substantial random variation.

As mentioned in Appendix 2.4 and Appendix 4.2, the number of considerations that potentially may need to be juggled when implementing the ACCE Method may seem daunting. However, this may partially reflect, at least in part, the lack of testing of the method that has thus far occurred, since the method has been just proposed. I would anticipate that as the method is implemented in simulated and real-world datasets, the relative importance of some of these components relative to others will become much clearer.

### 7.2. Potential advantages of multiple applications of the method using different Introduced Variables/Introduced Variable sets

One possibility that may emerge, especially as a better sense is developed of the impact of random variation and the strength of association with treatment exposure typically needed for reliable estimates, is that this method may perform best with multiple applications of the method featuring different Introduced Variables or Introduced Variable sets. These multiple applications of the method would obviously be in addition to any bootstrapping to obtain confidence intervals using the same Introduced Variable(s) (if this is the method settled upon for confidence interval generation). These multiple applications could either involve simply introducing different Introduced Variables to the same Model 1 or, probably a superior but more labor-intensive approach, to different Model 1s. This more labor-intensive approach would address the possibility that the correlated included and unmeasured confounders would likely vary from Introduced Variable to Introduced Variable, and because it may be desirable to include the other candidate Introduced Variables in the Model 1 when these variables are not being used as Introduced Variables. In the ideal scenario, even though each of these comparisons would involve at least slightly different measured covariates and, thus, different amounts of residual/unmeasured confounding in Model 1, the ACCE Method would yield the same, or close to the same, ultimate value for the unconfounded treatment effect.

If a similar estimate of the unconfounded treatment effect is not obtained, then one approach would be to choose the estimate that is most “conservative” (i.e., yields the lowest unconfounded treatment effect estimate). Whether in practice this might end up being too conservative (depending on the potential impacts of random variation) remains to be determined.

While the approach of applying this method multiple times is clearly more labor-intensive, in the era of Big Data, ample computer power, and automation, such an approach likely would be justified if it substantially improved the method’s estimate of unmeasured confounding. (The same consideration may apply to other potentially labor-intensive steps, such as estimating the residual confounding contributed by each of the included propensity score covariates [ Appendix 2.1 and Appendix Figure 1c]).

### 7.3. Final thoughts on the ACCE Method and the need for additional research

What has been outlined here is a method that, in theory, can derive a treatment effect estimate from nonrandomized studies less biased from residual baseline confounding, even when some of the residual confounding is unmeasured. It is even possible that in some cases this approach may also permit derivation of a treatment effect estimate that is less biased from confounding arising after treatment initiation as well ( Appendix 2.3b). The logic of the method is relatively straightforward. In addition, its core principle that introducing a predictable amount amplification of residual confounding should permit estimation (through extrapolation) of the amount of confounding prior to amplification may have enduring value, even if the specifics of the method outlined here are altered by future research. What remains to be determined is how well the method actually works in practice, when applied to simulated and real-world data.

It is difficult to predict the method’s performance *a priori*. While many challenges can be anticipated, further study of this method appears clearly warranted. Importantly, some of the many potential challenges anticipated in this manuscript would be expected to reduce the *accuracy* of the proposed method but not invalidate the basic approach. It also should be noted that the potential exists for this method to provide several distinct benefits: 1) the potential to use a greater variety of variables than strict instrumental variables as the basis to address unmeasured confounding (specifically, being able to use variables to that have associations with outcome, as well as those that do not); 2) the apparent potential to use a set of variables to produce confounding amplification when no single variable is sufficient, and 3) possibly the potential to address confounding after treatment initiation as well as at baseline. Thus, this proposed method seems to have enough potential advantages to justify its further investigation as one approach that might contribute to the broad aim of making nonrandomized treatment effect estimates less subject to confounding. Such research would also help establish whether this method also might contribute to the more challenging objective of deriving nonrandomized treatment effect estimates that truly approximate those that would be observed in randomized trials in that population.

For all these reasons it is hoped that the extent and the detail of the information provided here will allow research on this proposed method to proceed rapidly. Such research would allow for the important determination to be made regarding where this method stands in value compared to other methods proposed for obtaining unconfounded treatment effects.

Summary Equation for the ACCE Method ^h, i^

$$TEE_{M\text{1}}-\left(\frac{{(\text{TEE}}_{\text{M2}}-{\text{TEE}}_{\text{M1}})-{\text{Conf}}_{{\text{IntV}}_{\Delta \text{(M2}-\text{M1)}}}}{\left(\left(\frac{\frac{1}{\left(1-R_{\text{M2}}^{\text{2}}\right)}}{\frac{1}{\left(1-R_{\text{M1}}^{\text{2}}\right)}}\right)-1\right)}\right)-{\text{Conf}}_{{\text{IntV}}_{\text{M1}}}=\begin{array}{c} Unconfounded\\ TEE \end{array}$$

^a^”Internal Marker” = a measured covariate deliberately not included in the propensity score that is generally uncorrelated with other propensity score covariates. The internal marker serves to index the amount of confounding amplification between treatment groups that occurs between Model 1 and Model 2. If an internal marker is used, then Confounding Amplification (Camp) = Final Internal Marker Covariate Imbalance / Initial Internal Marker Covariate Imbalance.

^b^For all associations involving the Introduced Variable, the association would include the association of the Introduced Variable plus the associations of its correlates, to the extent that these associations influence the observed association between the Introduced Variable and outcome. When balance in the Introduced Variable is referenced, this also refers to balance in both the Introduced Variable and, to a lesser extent, its correlates.

^c^Either within-treatment arm or overall regressions can be performed. Examining the association within treatment arms prevents the association with the intervention, which may be substantial, from influencing the estimation of the IntV-Outcome association. The association between Introduced Variable and Outcome is an aggregate of direct and indirect associations. This aggregate association is then used in Step 3b to estimate the quantitative effect on the treatment effect estimate of adding the Introduced Variable into the propensity score (and increasing its balance through stratification or matching) that is independent of confounding amplification.

^d^Based on averaging the coefficients (i.e., ln(RR)). The most straightforward circumstance for the within-treatment arm approach is if the observed association is highly comparable in both treatment arms and both models. If so, as an approximation these values can be averaged. Determining these Introduced Variable-outcome regression coefficients separately for each model has an essential function. In Model 2, the Introduced Variable-outcome association would also be expected to suffer some confounding amplification relative to Model 1. In this hypothetical example, the observed association is varied slightly to illustrate that ( Appendix 4).

^e^Since the Introduced Variable(s) is a measured covariate or covariates, it is possible to determine its initial imbalance in Model 1, and the degree to which this imbalance changes in Model 2. This information can then be combined (through use of the Bross equation ^8^) with the coefficients derived in Step 3a to estimate the component of the change in the Treatment Effect Estimate between Model 1 and Model 2 that is attributable to increased balance in the Introduced Variable(s) and its correlates.

^f^The congruence between the scenario’s genuine treatment effect and total confounding and these values should not be seen as validating the method. The scenario’s effects estimates were selected based upon what would be expected from confounding amplification operating consistent with the system described here. However, this step-by-step example does illustrate how, absent the effects of random variability, the mechanics of how this series of calculations would function to produce the desired values (i.e., Total Residual Confounding and an Unconfounded Treatment Effect Estimate) from the initial values (i.e., the confounded Model 1 and 2 treatment effect estimates, an estimate of confounding amplification, and an estimate of the contribution to confounding of the Introduced Variable and, to a more uncertain degree, its correlates).

^g^To obtain the most rigorous estimate of the unconfounded treatment effect, in theory, two additional terms are necessary ( Appendix 2.1, Appendix 3.2a and Appendix Figure 1c). The first term estimates the contribution of any change in imbalance in the measured, included propensity score covariates from Model 1 to Model 2 to Model 2 residual confounding (as estimated through multivariate regression coefficients and the Bross equation) needs to be subtracted from the change in the treatment effect estimates (in addition to Confint delta). This subtraction is needed since this change in imbalance in the included propensity score covariates represents another process separate from full confounding amplification (i.e., to the degree predicted by R ^2^) that can contribute to the change in treatment effect estimate from Model 1 to Model 2. In general, I would expect the insertion of the Introduced Variable into Model 2 to typically worsen, at least slightly, any imbalance in the propensity score covariates that existed in Model 1. The second term represents the residual confounding in Model 1 attributable to the fact that the propensity score variables are not brought into perfect, 50/50 balance. The practical significance of these components in most instances is uncertain and these steps add considerable labor. Nevertheless, if these calculations are able to be performed routinely (perhaps by automating the process), this will ensure those instances where this adjustment is important will not be overlooked. Please see Appendix 2.1 and Appendix 3.2a for further discussion and Appendix Figure 1c for the more complex, more strictly rigorous, equation.

^h^If preferred, the equation can be written with a slightly simpler denominator:

$$TEE_{M\text{1}}-\left(\frac{{(\text{TEE}}_{M2}-{\text{TEE}}_{\text{M1}})-{\text{Conf}}_{{\text{IntV}}_{\Delta \text{(M2}–\text{M1)}}}}{\left(\left(\frac{\left(1-R_{\text{M1}}^{\text{2}}\right)}{\left(1-R_{\text{M2}}^{\text{2}}\right)}\right)-1\right)}\right)-{\text{Conf}}_{{\text{IntV}}_{\text{M1}}}=\begin{array}{c} Unconfounded\\ TEE \end{array}$$

However, I emphasize the slightly more elaborate form in this manuscript for two reasons. First, I think it makes the less reduced form of the equation makes clearer why the reduced form of the equation has the form it does (i.e., why the fraction in the denominator simplifies to: $\frac{\left(1-R_{\text{M1}}^{\text{2}}\right)}{\left(1-R_{\text{M2}}^{\text{2}}\right)}$). Second, I also think the less reduced form of the question, which relates to the understandable concept of “the confounding amplification of Model 2 divided by the proportional confounding of Model 1” in straightforward fashion, makes the overall equation seem more straightforward and easily understood, and hopefully, easier to remember.

In addition, the equation could also be alternatively written as follows:

$$TEE_{M\text{1}}-\left(\left(\frac{{(\text{TEE}}_{\text{M2}}-{\text{TEE}}_{\text{M1}})-{\text{Conf}}_{{\text{IntV}}_{\Delta \text{(M2}–\text{M1)}}}}{\left(\left(\frac{\frac{1}{\left(1-R_{\text{M2}}^{\text{2}}\right)}}{\frac{1}{\left(1-R_{\text{M1}}^{\text{2}}\right)}}\right)-1\right)}\right)+{\text{Conf}}_{{\text{IntV}}_{\text{M1}}}\right)=\begin{array}{c} Unconfounded\\ TEE \end{array}$$

a form which emphasizes the fact that both terms subtracted from TEE _M1_ together constitute the sum of residual confounding.

^i^For the scenario posed at the beginning of the table, the method’s Summary Equation would arrive at the following estimate of the unconfounded treatment effect:

beta coefficient = 0.2351 - ((0.2612 - 0.2351) - (-0.0273)) / (((1 / (1 - 0.5)) / (1 / (1 - 0.25))) - 1) - 0.0293 = 0.0990 (unconfounded TEE) (exponentiated, the unconfounded TEE equals RR = 1.10).
