# Supplementary material for: The ACCE method: an approach for obtaining quantitative or qualitative estimates of residual confounding that includes unmeasured confounding
Source: F1000Res. 2015 Apr 29;3:187. Originally published 2014 Aug 11. [Version 2] doi: 10.12688/f1000research.4801.2 (PMC4288424; doi:10.12688/f1000research.4801.2)
Supplement: Peer review history

# Peer review: Referee response for version 1

**DOI:** 10.5256/f1000research.5125.r7091

**Published:** 2015-01-05

**Authors:** Gregory Matthews

**Affiliations:** 1 Department of Mathematics and Statistics, Loyola University Chicago, Chicago, IL, USA

The authors present a manuscript describing a procedure that allows for the quantification of the total amount of residual confounding prior to bias amplification caused by propensity score models.  I believe the procedure described in reasonable, and my biggest concerns with this manuscript are the presentation of the approach, which I had a hard time following initially. I think this paper is deserving of indexing as it is, but could be substantially improved with clearer presentation. 

Specific Comments:

- The authors talk about creating two models (Model 1 and Model 2) that are nest within each other in such a way that Model 2 contains all the variables in Model 1 plus one/several extra variable/s.  It seems like there are money choices for this extra variable/s from among the possible variables.  Do the authors have any specific advice on how this or these should be chosen?  They do mention that this variable should be chosen to have ``discernible confounding amplification", but isn't it possible that there are many acceptable choices that will satisfy this criteria?  In that case is there any advice on how to choose between the good candidate variables?  
- In Step 2 of the description of the method,the authors mention that the when $R^2$ is between 0.04 and 0.56 there is a linear relationship between unexplained variance and confounding amplification.  I believe that this threshold is then used in Supplementary table 1 when they state that the step should be taken only if R^2 is less than 0.56.  Should this step not be taken if R^2 is less than 0.04?  Do the authors have any advice on what to do when R^2 is greater than 0.56? 

Minor Comments:

- Should the outcome in Table 1B be hip fracture rather than all cause mortality?
- Supplementary Table1, 3a I think this is a typo: ``IntV:"

I have read this submission. I believe that I have an appropriate level of expertise to confirm that it is of an acceptable scientific standard.

# Response

**Published:** 2015-03-02

**Authors:** Eric Smith

**Affiliations:** ENRM VAMC Bedford, MA / Center for Healthcare Organization and Implementation Research / UMass Medical School Dept. of Psychiatry, USA

I would like to thank both reviewers for their thoughtful, insightful, and encouraging reviews.  I particular appreciate their openness to a new methodology to attempt to estimate residual/unmeasured confounding.  I am very glad to see that they recognized the value in disseminating and exploring a methodology that takes a very different approach (and possibly an approach that is more broadly applicable) than some of the limited number of alternatives currently available to tackle the problem of unmeasured confounding.  Their specific comments were also extremely valuable.

Both reviewers suggested that the manuscript would benefit from greater clarity; therefore I have revised and enhanced the presentation of the method quite substantially.  The major ways I have done this is to: 1) expand the description of the method in the text and adding cross-references to the exact steps in the Appendix Table (which has also been expanded); 2) adding 3 additional hypothetical examples to communicate more incrementally the rationale for the method; 3) reorganized the manuscript Table so it reads more vertically than horizontally; 4) attempted to be more precise and detailed in my language; and, perhaps most importantly, 5) expressed the entire method mathematically in a single Summary Equation to help facilitate its understanding.  The main manuscript text is substantially longer as a result of this increased explanation, but hopefully less ambiguous at key points.  Some of the increase in length results from the more detailed description of the method, but much of the increase relates to the more detailed hypothetical examples, which some readers may not even feel a need to review.  Similarly, the Appendices are considerably longer, but the reader is encouraged to pick and choose whether they want to review some, none, or all of these based entirely on their interest.

Another important comment was Dr. Lunt’s comment that considerably further work needed to be done on the method.  I couldn’t agree more, and it is my hope that the dividend that results from laying out the method in such detail is that multiple research groups can quickly advance this research.  As I try to anticipate and highlight as fully as possible, there are a number of important uncertainties.  These uncertainties range from such fundamental points as how consistently predictable the phenomenon of confounding amplification actually is, how accurately the difference between effect estimates can be determined, and how accurate are the proposed Bross equation-based corrections for the contribution of the Introduced Variable and, to a partial degree, its correlates, on the estimates of the change in treatment effect estimate as well as the starting Model 1 treatment effect estimate.  Indeed, it is not even certain whether the method can be applied to some common logistic model effect estimates (e.g., odds ratio).  I have even identified two more potential sources of uncertainty that are now included and discussed in the text and appendices: whether the introduced variable-outcome regression coefficient would potentially also suffer from at least some confounding amplification, and whether possible “constraints” might exist to achievable confounding amplification in real-world settings.  So I am in complete agreement with Dr. Lunt that this manuscript represents only the very start of what hopefully will be steady advance of knowledge about this method and its value relative to other proposed approaches addressing unmeasured confounding.  To my point of view, this is all the more reason to seek to enlist the greater research community in this effort.

Nevertheless, it is important to note that approaches suggest themselves to address or minimize many of these uncertainties, although much investigation is needed.  In addition, I want to emphasize a key point: while a number of uncertainties exist relevant to the actual performance of the method, it is my intention that, with this version of the manuscript, that there be no substantial uncertainty concerning the specific approach that is actually being proposed.  I paid close attention to the fact that Dr. Matthews and Dr. Lunt (who has published on bias amplification) appeared uncertain about how to apply the method as described in Version 1.  I hope in this version that I have communicated the method clearly enough that the vital next step can take place: testing the method in simulated and real-world datasets.

It is for this reason – to facilitate the ability of as many interested research teams as possible to contribute to the method’s evaluation and evolution – that I have taken particular pains to expand communication concerning the overall logic, and underlying rationale, of the method and each of its steps. There are certainly places in which my proposed solutions to potential challenges for the method may prove imperfect or suboptimal (some possibilities might include the use of a regression coefficient and the Bross equation to take account confounding from the Introduced Variable-outcome relationship, the suggested approach to addressing possible confounding amplification in the Introduced Variable-outcome coefficient, and/or the favoring of stratification over matching to increase comparability of Model 1 and Model 2 mentioned in Appendix 2).  It is my firm hope that other research groups can contribute by suggesting other approaches to accomplishing that particular objective within in the method, or even other angles concerning how to exploit confounding amplification to help estimate residual confounding. Therefore I wanted to be particularly clear in explaining the method so that the objective to be accomplished in each step was clear.  This communication has been done through expanded text, calculations, examples, metaphors, technical Appendices, and the Summary Equation. I also outline the clear initial and subsequent steps for research as I see them (most centered on simulation) in the Discussion.  Hopefully the manuscript is now sufficiently clearer so that collaborative investigation and elaboration of this method can take place.

I thank the reviewers for encouraging me to much more carefully clarify the logic and approach of the method, and I hope they think that I have succeeded in that task.

In closing, I would like to address the remaining specific points brought up by the reviewers:

Dr. Lunt (Reviewer 1):

 

1. As mentioned above, I am extremely grateful for Dr. Lunt’s observation for noting that the denominator of equations 3-6 in Reference 4 (Pearl, 2011) does indeed appear to support the 1-R ^2^ relationship predicting the proportional amount of confounding amplification separate from the Brooks and Ohsfeldt (2013) simulation.  This is potentially quite important, for it suggests that application of the technique might not need to be limited to an R ^2^ of ≤ 0.56 (one of the concerns of the 2 ^nd^ reviewer, Dr. Matthews).  It does, however, increase the need to understand why the Brooks and Ohsfeldt simulation begins to exhibit nonlinear confounding amplification above R ^2^ of 0.56.

 

Dr. Matthews (Reviewer 2):

 

1. Dr. Matthews asked a number of helpful questions concerning important details involved in implementing the method that I see now were not addressed as directly and thoroughly as they might have been.  So that many readers can easily benefit from his helpful inquiry concerning recommendations on how to choose an instrumental variable without having to access my response to this comment, I have added an entire Appendix (Appendix 7) devoted in large part to this topic.  In addition to offering practical suggestions on implementing the method, based on current knowledge, this Appendices also attempts to anticipate the likely trade-offs involved in optimizing one characteristic of the method potentially at the cost of another characteristic (e.g., wanting to maximize confounding amplification while minimizing differences between the two models that are separate from confounding amplification).
2. Regarding Dr. Matthew’s 2 ^nd^ major point, the simulation research that I hope follows this manuscript will likely provide the best guidance on what approaches should be taken if the R ^2^ is < 0.04 or > 0.56.  It should be noted, however, that, until that research is available, it is to be hoped that almost all propensity score models will succeed in achieving an R ^2^ of at least 0.04.  Furthermore, one remedy for circumstances in which Model 2 exceeds an R ^2^ of 0.56 seemingly would be simply to remove measured covariates from the propensity score model until Model 2’s R2 is ≤ 0.56.  This is a pragmatic, but not a perfect solution, since as pointed out in Appendix 3.2, such a step places extra weight on the method achieving an accurate estimate of residual/unmeasured confounding, since more of that type of confounding now exists.  Also, as discussed in Appendix 4, if variables have to be removed from the propensity score, priority should be given to removing variables with little or no correlation with the Introduced Variable(s) and retaining in the propensity scores, to the extent possible, variables that correlate with the Introduced Variable(s) 
3. I also thank Dr. Matthews for pointing out the mislabeling of the outcome in Table 1.  As mentioned, in addition to correcting this error, I have entirely restructured this Table to make it read more vertically than horizontally, at least in regard to the information pertaining to Model 1 versus Model 2. 
4. Regarding the “IntV” terminology in Supplementary Table 1, I have retained this abbreviation. “IntV” is my attempt to propose a nomenclature (abbreviation) for the introduced variable that will separate it from instrumental variables (which, unfortunately, share the same initials).  “InV” might also be useable, but I felt the extra letter of “IntV” as an abbreviation for the term “Introduced Variable” made sense because the abbreviation was less likely to appear to be simply an erroneous typing of “IV.”  

I have also made the following minor changes:

 

1. Capitalized “Introduced Variable(s)” to make each of its mentions more noticeable, since this variable or variables plays a key role in the method.
2. Expanded the discussion of the potential impacts of correlations between various types of variables on the method’s estimates, and added Appendices that explore potential threats to the accuracy of the Introduced Variable-outcome regression coefficient, that provide explanation of the method’s components (and key uncertainties) in reference to the terms of the ACCE Method Summary Equation, and that begin to explore the use of sets of Introduced Variables and the practical trade-offs to be considered when implementing the method.
3. Tried to be consistent with my language concerning “confounding amplification”:  “proportional confounding amplification” refers to the percentage increase in residual confounding predicted by 1-R2, some other measure of exposure prediction, or an internal marker, while “quantitative confounding amplification” refers to the numerical change in the treatment effect estimate (technically, the change in the treatment effect estimate adjusted for the impact of increased balance in the Introduced Variable(s)). 
4. Replaced the term “multiple” Introduced Variable(s) with the term “set of Introduced Variables” to make it clearer I am referring to simultaneously insertion of several to many Introduced Variables, rather than the sequential use of different single Introduced Variables.
5. Clearly labeled the Hypothetical Examples as Hypothetical Examples, moving them out of “Results.”
6. Changed the examples from “odds ratio” to “risk ratio” due to concerns that noncollapsibility of the odds ratio might interfere with the subtraction of the Model 1 and Model 2 treatment effect estimates necessary to estimate the quantitative effect of confounding amplification.
7. Invented the term “amplifiable fraction of residual confounding” to hopefully better communicate that (if the Introduced Variable(s) has any association with outcome) it is only the residual confounding separate from that which is attributable to the Introduced Variable(s) (which is not amplified) that is able to be amplified.  Hopefully this has made this clearer.
8. Removed the somewhat redundant word “Supplementary” from “Supplementary Appendix Table.”
9. Corrected a minor subtraction error in the Appendix Table, Equation 3b (and subsequent steps), that had no substantive impact on the estimates of total residual confounding and the unconfounded treatment effect estimate. Also corrected a notation error in Step 4a where “M2” had been written “M3” by mistake.

# Peer review: Referee response for version 1

**DOI:** 10.5256/f1000research.5125.r6843

**Published:** 2014-11-27

**Authors:** Mark Lunt

**Affiliations:** 1 Arthritis Research UK Epidemiology Unit, University of Manchester, Manchester, UK

This article outlines a very interesting approach to using propensity score methods to correct for unmeasured confounding. That was not the aim of the propensity score, and current methods are not able to do this, so it potentially represents a considerable advance.

The idea is conceptually a simple one, related to the well-established use of instrumental variables to control for unmeasured confounding. However, I have not come across this idea before, and the author is to be congratulated on his originality.

Having said that, I was a little disappointed in the presentation of the method. I do not feel that I am in a position to apply this method to any of my own data. One reason that I took so long over the review was that I wanted to be certain I fully understood the method by applying it myself, but it has become obvious that I will not be able to in a reasonable timescale.

Greater precision in the presentation would have been welcome, whether that was explicit mathematical formulae, or simply causal diagrams showing how the various biases arose and which causal paths contributed to which estimates. This has been done very well in some of the references. For example, the relation between bias amplification and 1-R ^2^ is given a clear mathematical basis in reference 4, and I would regard this as more convincing than simulation evidence.

I’m sure that the author would agree with me that there is a lot of work to be done on this method before it can be applied routinely. I hope that this paper does spark that research, and that the author gets the credit he deserves for coming up with this potentially very useful idea.

I have read this submission. I believe that I have an appropriate level of expertise to confirm that it is of an acceptable scientific standard.

# Response

**Published:** 2015-03-02

**Authors:** Eric Smith

**Affiliations:** ENRM VAMC Bedford, MA / Center for Healthcare Organization and Implementation Research / UMass Medical School Dept. of Psychiatry, USA

I would like to thank both reviewers for their thoughtful, insightful, and encouraging reviews.  I particular appreciate their openness to a new methodology to attempt to estimate residual/unmeasured confounding.  I am very glad to see that they recognized the value in disseminating and exploring a methodology that takes a very different approach (and possibly an approach that is more broadly applicable) than some of the limited number of alternatives currently available to tackle the problem of unmeasured confounding.  Their specific comments were also extremely valuable.

Both reviewers suggested that the manuscript would benefit from greater clarity; therefore I have revised and enhanced the presentation of the method quite substantially.  The major ways I have done this is to: 1) expand the description of the method in the text and adding cross-references to the exact steps in the Appendix Table (which has also been expanded); 2) adding 3 additional hypothetical examples to communicate more incrementally the rationale for the method; 3) reorganized the manuscript Table so it reads more vertically than horizontally; 4) attempted to be more precise and detailed in my language; and, perhaps most importantly, 5) expressed the entire method mathematically in a single Summary Equation to help facilitate its understanding.  The main manuscript text is substantially longer as a result of this increased explanation, but hopefully less ambiguous at key points.  Some of the increase in length results from the more detailed description of the method, but much of the increase relates to the more detailed hypothetical examples, which some readers may not even feel a need to review.  Similarly, the Appendices are considerably longer, but the reader is encouraged to pick and choose whether they want to review some, none, or all of these based entirely on their interest.

Another important comment was Dr. Lunt’s comment that considerably further work needed to be done on the method.  I couldn’t agree more, and it is my hope that the dividend that results from laying out the method in such detail is that multiple research groups can quickly advance this research.  As I try to anticipate and highlight as fully as possible, there are a number of important uncertainties.  These uncertainties range from such fundamental points as how consistently predictable the phenomenon of confounding amplification actually is, how accurately the difference between effect estimates can be determined, and how accurate are the proposed Bross equation-based corrections for the contribution of the Introduced Variable and, to a partial degree, its correlates, on the estimates of the change in treatment effect estimate as well as the starting Model 1 treatment effect estimate.  Indeed, it is not even certain whether the method can be applied to some common logistic model effect estimates (e.g., odds ratio).  I have even identified two more potential sources of uncertainty that are now included and discussed in the text and appendices: whether the introduced variable-outcome regression coefficient would potentially also suffer from at least some confounding amplification, and whether possible “constraints” might exist to achievable confounding amplification in real-world settings.  So I am in complete agreement with Dr. Lunt that this manuscript represents only the very start of what hopefully will be steady advance of knowledge about this method and its value relative to other proposed approaches addressing unmeasured confounding.  To my point of view, this is all the more reason to seek to enlist the greater research community in this effort.

Nevertheless, it is important to note that approaches suggest themselves to address or minimize many of these uncertainties, although much investigation is needed.  In addition, I want to emphasize a key point: while a number of uncertainties exist relevant to the actual performance of the method, it is my intention that, with this version of the manuscript, that there be no substantial uncertainty concerning the specific approach that is actually being proposed.  I paid close attention to the fact that Dr. Matthews and Dr. Lunt (who has published on bias amplification) appeared uncertain about how to apply the method as described in Version 1.  I hope in this version that I have communicated the method clearly enough that the vital next step can take place: testing the method in simulated and real-world datasets.

It is for this reason – to facilitate the ability of as many interested research teams as possible to contribute to the method’s evaluation and evolution – that I have taken particular pains to expand communication concerning the overall logic, and underlying rationale, of the method and each of its steps. There are certainly places in which my proposed solutions to potential challenges for the method may prove imperfect or suboptimal (some possibilities might include the use of a regression coefficient and the Bross equation to take account confounding from the Introduced Variable-outcome relationship, the suggested approach to addressing possible confounding amplification in the Introduced Variable-outcome coefficient, and/or the favoring of stratification over matching to increase comparability of Model 1 and Model 2 mentioned in Appendix 2).  It is my firm hope that other research groups can contribute by suggesting other approaches to accomplishing that particular objective within in the method, or even other angles concerning how to exploit confounding amplification to help estimate residual confounding. Therefore I wanted to be particularly clear in explaining the method so that the objective to be accomplished in each step was clear.  This communication has been done through expanded text, calculations, examples, metaphors, technical Appendices, and the Summary Equation. I also outline the clear initial and subsequent steps for research as I see them (most centered on simulation) in the Discussion.  Hopefully the manuscript is now sufficiently clearer so that collaborative investigation and elaboration of this method can take place.

I thank the reviewers for encouraging me to much more carefully clarify the logic and approach of the method, and I hope they think that I have succeeded in that task.

In closing, I would like to address the remaining specific points brought up by the reviewers:

Dr. Lunt (Reviewer 1):

 

1. As mentioned above, I am extremely grateful for Dr. Lunt’s observation for noting that the denominator of equations 3-6 in Reference 4 (Pearl, 2011) does indeed appear to support the 1-R ^2^ relationship predicting the proportional amount of confounding amplification separate from the Brooks and Ohsfeldt (2013) simulation.  This is potentially quite important, for it suggests that application of the technique might not need to be limited to an R ^2^ of ≤ 0.56 (one of the concerns of the 2 ^nd^ reviewer, Dr. Matthews).  It does, however, increase the need to understand why the Brooks and Ohsfeldt simulation begins to exhibit nonlinear confounding amplification above R ^2^ of 0.56.

 

Dr. Matthews (Reviewer 2):

 

1. Dr. Matthews asked a number of helpful questions concerning important details involved in implementing the method that I see now were not addressed as directly and thoroughly as they might have been.  So that many readers can easily benefit from his helpful inquiry concerning recommendations on how to choose an instrumental variable without having to access my response to this comment, I have added an entire Appendix (Appendix 7) devoted in large part to this topic.  In addition to offering practical suggestions on implementing the method, based on current knowledge, this Appendices also attempts to anticipate the likely trade-offs involved in optimizing one characteristic of the method potentially at the cost of another characteristic (e.g., wanting to maximize confounding amplification while minimizing differences between the two models that are separate from confounding amplification).
2. Regarding Dr. Matthew’s 2 ^nd^ major point, the simulation research that I hope follows this manuscript will likely provide the best guidance on what approaches should be taken if the R ^2^ is < 0.04 or > 0.56.  It should be noted, however, that, until that research is available, it is to be hoped that almost all propensity score models will succeed in achieving an R ^2^ of at least 0.04.  Furthermore, one remedy for circumstances in which Model 2 exceeds an R ^2^ of 0.56 seemingly would be simply to remove measured covariates from the propensity score model until Model 2’s R2 is ≤ 0.56.  This is a pragmatic, but not a perfect solution, since as pointed out in Appendix 3.2, such a step places extra weight on the method achieving an accurate estimate of residual/unmeasured confounding, since more of that type of confounding now exists.  Also, as discussed in Appendix 4, if variables have to be removed from the propensity score, priority should be given to removing variables with little or no correlation with the Introduced Variable(s) and retaining in the propensity scores, to the extent possible, variables that correlate with the Introduced Variable(s) 
3. I also thank Dr. Matthews for pointing out the mislabeling of the outcome in Table 1.  As mentioned, in addition to correcting this error, I have entirely restructured this Table to make it read more vertically than horizontally, at least in regard to the information pertaining to Model 1 versus Model 2. 
4. Regarding the “IntV” terminology in Supplementary Table 1, I have retained this abbreviation. “IntV” is my attempt to propose a nomenclature (abbreviation) for the introduced variable that will separate it from instrumental variables (which, unfortunately, share the same initials).  “InV” might also be useable, but I felt the extra letter of “IntV” as an abbreviation for the term “Introduced Variable” made sense because the abbreviation was less likely to appear to be simply an erroneous typing of “IV.”  

I have also made the following minor changes:

 

1. Capitalized “Introduced Variable(s)” to make each of its mentions more noticeable, since this variable or variables plays a key role in the method.
2. Expanded the discussion of the potential impacts of correlations between various types of variables on the method’s estimates, and added Appendices that explore potential threats to the accuracy of the Introduced Variable-outcome regression coefficient, that provide explanation of the method’s components (and key uncertainties) in reference to the terms of the ACCE Method Summary Equation, and that begin to explore the use of sets of Introduced Variables and the practical trade-offs to be considered when implementing the method.
3. Tried to be consistent with my language concerning “confounding amplification”:  “proportional confounding amplification” refers to the percentage increase in residual confounding predicted by 1-R2, some other measure of exposure prediction, or an internal marker, while “quantitative confounding amplification” refers to the numerical change in the treatment effect estimate (technically, the change in the treatment effect estimate adjusted for the impact of increased balance in the Introduced Variable(s)). 
4. Replaced the term “multiple” Introduced Variable(s) with the term “set of Introduced Variables” to make it clearer I am referring to simultaneously insertion of several to many Introduced Variables, rather than the sequential use of different single Introduced Variables.
5. Clearly labeled the Hypothetical Examples as Hypothetical Examples, moving them out of “Results.”
6. Changed the examples from “odds ratio” to “risk ratio” due to concerns that noncollapsibility of the odds ratio might interfere with the subtraction of the Model 1 and Model 2 treatment effect estimates necessary to estimate the quantitative effect of confounding amplification.
7. Invented the term “amplifiable fraction of residual confounding” to hopefully better communicate that (if the Introduced Variable(s) has any association with outcome) it is only the residual confounding separate from that which is attributable to the Introduced Variable(s) (which is not amplified) that is able to be amplified.  Hopefully this has made this clearer.
8. Removed the somewhat redundant word “Supplementary” from “Supplementary Appendix Table.”
9. Corrected a minor subtraction error in the Appendix Table, Equation 3b (and subsequent steps), that had no substantive impact on the estimates of total residual confounding and the unconfounded treatment effect estimate. Also corrected a notation error in Step 4a where “M2” had been written “M3” by mistake.
